# Unraveling the Complex Hydrogen-Bonding and Solvation Patterns of Fructose Tautomers via Complementary AIMD and Classical MD Simulations

**DOI:** 10.1021/acsphyschemau.6c00122

**Published:** 2026-09-08

**Authors:** Imre Bakó, Szilvia Pothoczki

**Affiliations:** † HUN-REN Research Centre for Natural Sciences, Magyar tudósok körútja 2, Budapest H-1117, Hungary; ‡ HUN-REN Wigner Research Centre for Physics, Konkoly-Thege M. Út 29-33, Budapest H-1121, Hungary

**Keywords:** carbohydrate solvation, classical molecular dynamics simulation, ab initio molecular dynamics simulations, H-bonding, Bader charges, dipole moment

## Abstract

Understanding the molecular-level hydration of fructose tautomers remains a challenge due to their rapid interconversion and structural complexity. A dual-scale approach was employed, combining long-time classical molecular dynamics (MD) and ab initio molecular dynamics (AIMD) to elucidate differences in the hydrogen-bonding networks of both pyranose and furanose tautomers with statistical and electronic accuracy. Our simulations highlight the inherent competition between intra- and intermolecular hydrogen bonds, driven by the limited number of hydroxyl donor and acceptor sites. Crucially, the formation of these internal bonds is driven by ring puckering in the flexible furanoses alongside the spatial orientation of the hydroxymethyl groups in both tautomer families. This structural interplay indirectly influences the entire intermolecular solvation shell, comprising both hydrophilic and hydrophobic regions. By correlating these competing intra- and intermolecular patterns with Bader charges and water dipole moments, we establish a quantitative electronic-structure basis for carbohydrate solvation. These insights provide a comprehensive atomistic framework that deepens our understanding of biologically relevant processes, such as recognition and docking efficiency within metabolic enzymes.

## Graphical abstract

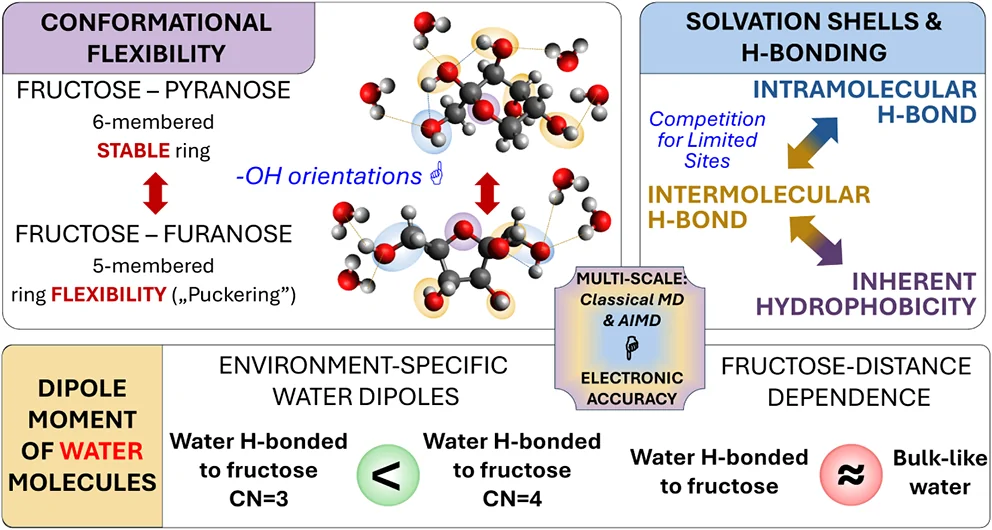

## Introduction

Understanding the behavior of fructose in aqueous solutions is crucial, as water facilitates continuous equilibrium transitions between its structural forms. This structural flexibility ensures that sugar can adapt to different chemical environments, which directly influences how it is recognized and processed by various biological systems.^1–3^ The solute-solvent interactions in aqueous solutions of fructose are fundamentally complex, involving a delicate balance between configurational and conformational equilibria of the solute molecule’s various forms.^4^ Configurational interconversion involves the transformation between the acyclic (linear) form of fructose and its cyclic (ring) forms, which can be five-membered (furanose) or six-membered (pyranose). Ring closure creates a new chiral center at the anomeric carbon, resulting in the formation of α and β anomers. Consequently, in aqueous solution, fructose exists in a dynamic tautomeric equilibrium among five forms; at 20 °C in D_2_O, the distribution is dominated by β-d-fructopyranose (68.23%) and β-d-fructofuranose (22.35%), with smaller amounts of α-d-fructofuranose (6.24%), α-d-fructopyranose (2.67%), and a minor open-chain fraction (0.50%).^3^ Conformational point of view: in aqueous solution, the molecule twists and “flips” without breaking any bonds. The dominant pyranose form of fructose maintains a chair conformation to minimize internal strain by keeping its bulky hydroxyl groups in stable positions.^5,6^ On the other hand, the more flexible furanose rings lack a single fixed shape and undergo constant “pseudorotation”, a restless flexing through various envelope and twist shapes that prevents them from settling into a single rigid geometry.^7–9^ This so-called puckering of the ring is biologically vital, for example, dictating how effectively the sugar docks onto metabolic enzymes.^10–13^

This significant biological activity, together with its high solubility, is largely due to fructose’s five hydroxyl groups and its ring oxygen, which provides extensive hydrogen-bonding (H-bonding) potential. There is competition between intramolecular H-bonds and intermolecular interactions with water for these available bonding sites.^4,14–16^ In addition to the number of hydroxyl groups, their relative orientations also play an important role.

The physicochemical characterization of aqueous fructose solutions has been addressed using a wide range of experimental and computational approaches.^14–22^ Structural insights have primarily been obtained using X-ray^14^ and neutron^15^ diffraction techniques. Insights into water dynamics and solute-solvent interactions are provided by terahertz time-domain attenuated total reflection spectroscopy^16^ and dielectric relaxation spectroscopy.^17^ From a computational perspective, molecular dynamics (MD) simulations have emerged as a critical tool for examining structural and dynamical behavior,^18–20^ alongside quantum-chemical calculations using density-functional theory.^21^ Multiple studies provide specific results regarding H-bonding;^15,16,18,22^ however, it must be emphasized that parameters such as the number or length of the bonds depend strongly on the H-bond definition and the experimental or computational method employed. Furthermore, a direct comparison is often hindered by the fact that most existing studies evaluate sugars as equilibrium tautomeric mixtures.

However, several critical questions regarding the local H-bonding environment remain unresolved; to bridge this gap, we employ a dual-scale computational strategy. In the first stage, classical Molecular Dynamics (MD) simulations were performed to explore the configurational space and provide sufficient configurational sampling over nanosecond time scales for both furanose and pyranose tautomers in aqueous environments. The corresponding molecular representations and atom-numbering scheme are detailed in Figure 1. From the resulting statistical ensembles, representative snapshots capturing key orientational states of the exocyclic hydroxymethyl group were extracted. These configurations then served as the starting points for ab initio Molecular Dynamics (AIMD) simulations. The AIMD models are utilized to accurately describe the electronic structure and the highly directional nature of H-bonding. This integrated approach allows for a first-principles description of the solute-solvent interactions—explicitly accounting for polarization effects and charge transfer—while ensuring that the analyzed structures are statistically representative of the broader conformational landscape. In the following sections, we evaluate the results from both simulation methods, combining the long-time statistical insights of classical MD with the electronic-level accuracy of AIMD. This approach was effective in our previous research on monosaccharides with pyranose rings.^23^

**Figure 1:**
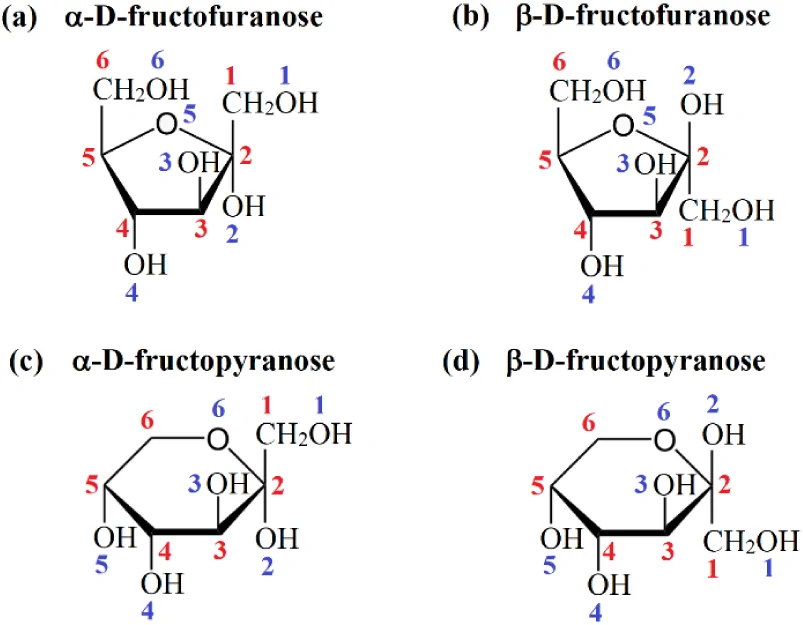
Haworth projections and atom numbering used in the furanose and pyranose structures of fructose: (a) α-d-fructofuranose, (b) β-d-fructofuranose, (c) α-d-fructopyranose, and (d) β-d-fructopyranose. Atom numbering: blue and red colors represent oxygen and carbon atoms, respectively.

Primarily, the precise role of the orientation of the exocyclic hydroxymethyl group in solute-solvent interactions has yet to be fully elucidated. Our first aim is to address the specific impact of the exocyclic hydroxymethyl group’s orientation on the H-bonding network within aqueous fructose solutions in both the furanose and pyranose forms.

The hydroxyl groups and the ring oxygen of the fructose molecule provide a limited number of H-bond donor and acceptor sites. When a site is involved in an intramolecular bond, it becomes unavailable for interaction with the surrounding water. We aim to analyze both intra- and intermolecular bonding patterns simultaneously to reveal how their competition shapes the H-bond network.

However, not all water molecules in the vicinity of the fructose molecule are involved in H-bonding. Along with the hydrophilic shell, there is also a hydrophobic layer that contains water molecules situated closer to the molecule’s carbon–hydrogen backbone.^24–27^ In these regions, where conventional hydrogen bonding is absent, the water molecules interact with the sugar through weaker van der Waals forces and CH···O_water_ type interactions. Simultaneously, however, they remain fully integrated into the solvent’s structure by forming H-bonded networks with neighboring water molecules.^24^ Consequently, we pay particular attention to characterizing their properties to understand the entire hydration environment.

We further aim to evaluate how fructose affects both the electronic and H-bonding properties of the solvent, specifically comparing water molecules directly bonded to the solute with those in the bulk-like region. The novelty of this work lies in linking the electronic properties of water molecules to their specific H-bonding states. Specifically, we calculate dipole moments for individual water molecules, categorized by the number of hydrogen bonds they form.

Another novelty of this work is its quantitative description of the water distribution around the fructose molecule. Based on our previous study of glucose,^23^ we have expanded the analysis from a simple *Z*-directional view (above and below the molecular plane) to a comprehensive three-dimensional (XYZ) spatial characterization. This approach is driven by the complex molecular geometry of fructose, which requires a full XYZ mapping to accurately describe its hydration shell. Finally, based on our findings, we attempt to provide insights into why β-pyranose remains the most abundant species in aqueous environments.

## Results

### Conformational Analysis

The conformation of cyclic fructose forms, whether fructopyranose (6-membered ring) or fructofuranose (5-membered ring), is characterized by the appropriate dihedral angles or dihedral angle pairs of the exocyclic hydroxymethyl group or hydroxide groups at anomeric C2. Additionally, for the furanose ring, the puckering parameters quantify the ring’s non-planarity. The stability of these conformations is greatly affected by competition among steric effects, anomeric interactions, dipole–dipole interactions, and intramolecular hydrogen bonding.

The key dihedral angles determining the conformation are the C2–C1–O1–H1, C5–C6–O6–H6, O5–C2–C1–O1, and O5–C5–C6–O6 for fructofuranose; and C2–C1–O1–H1, O6–C2–O2–H2, and O6–C2–C1–O1 for fructopyranose. The distributions of these dihedral angles, calculated from classical MD simulations, are shown in Figure 2, where *g* ^*+*^ and *g* ^–^ correspond to clockwise and counterclockwise *gauche* orientations, respectively, and *t* to the *trans* orientation. The applied atom numbering for both the pyranose and furanose structures of d-fructose is shown in Figure 1. Almost all monitored dihedral angles predominantly occur in the *g* ^+^ and *g* ^–^ conformations. However, for β-d-fructopyranose (Figure 2d), *t* conformations also appear with significant probability for the O6–C2–O2–H2 dihedral angle. Besides, for the other investigated dihedral angles, the occurrence of the *t* form is less than 20%. In the case of α-d-fructofuranose (Figure 2a), the O5–C2–C1–O1 and the O5–C5–C6–O6 dihedral angles show a preference for *g* ^–^. But this latter one is less pronounced. In this dual *g* ^–^ state, both hydroxymethyl groups turn toward the ring oxygen (O5) from opposite faces of the ring. Specifically, C6 folds back toward O5 from above, while C1 rotates toward it from below. For β-d-fructofuranose (Figure 2b), the distribution of these two dihedral angles is more symmetrical.

**Figure 2:**
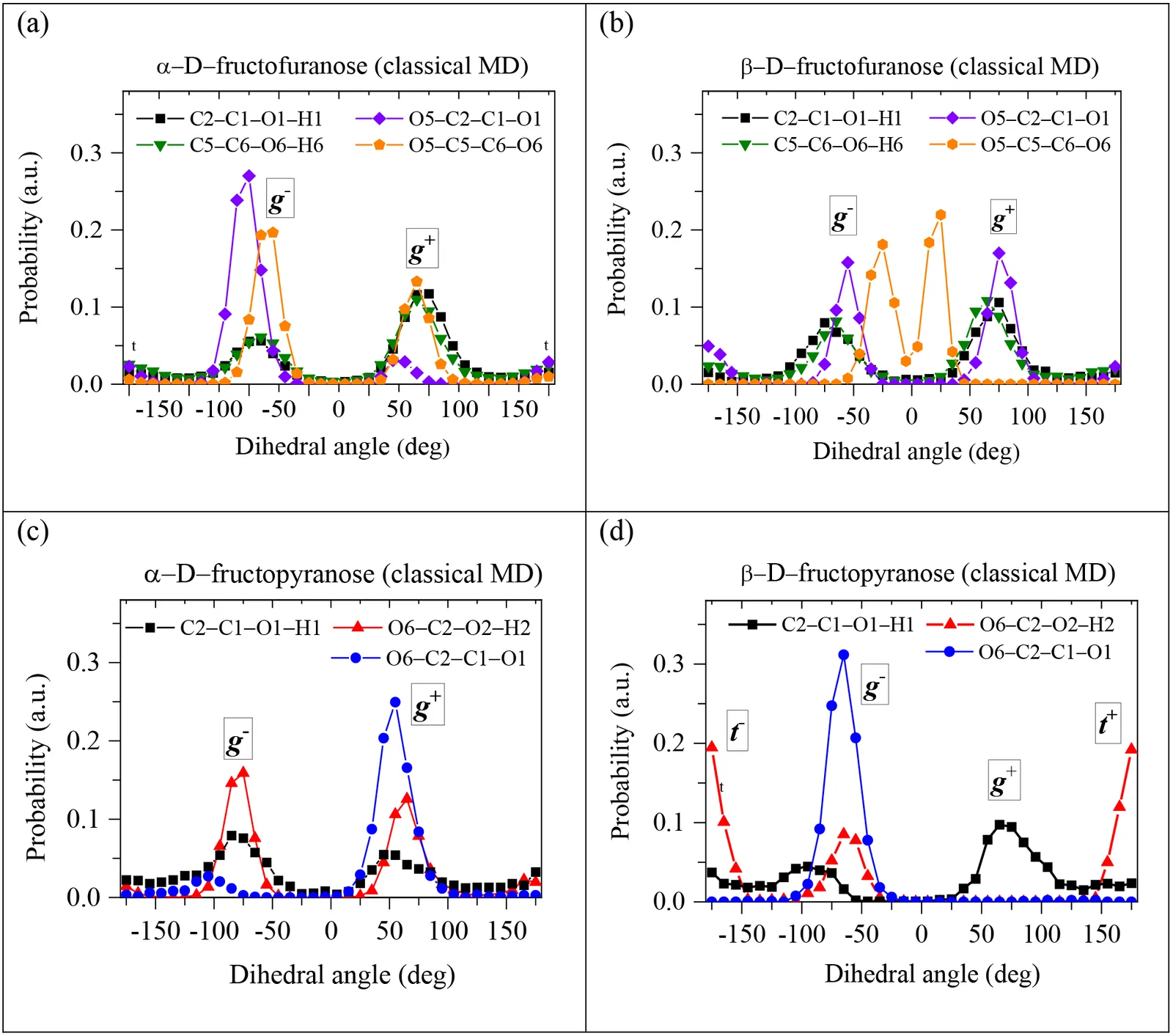
Distribution of dihedral angles from classical MD simulation for (a) α-d-fructofuranose, (b) β-d-fructofuranose, (c) α-d-fructopyranose, (d) β-d-fructopyranose.

For the two fructopyranose systems (Figure 2c and Figure 2d), the O6–C2–C1–O1 dihedral angle indicates the almost exclusive presence of the *g* ^+^ population for the α-anomer, with the hydroxymethyl group rotating inward toward ring oxygen (O6), and the *g* ^–^ state for the β-anomer, which points away from the ring.

Table 1 presents the differences between classical MD and AIMD models regarding characteristic conformations defined by the introduced dihedral angles. For this analysis, three representative furanose (AFF1–3 and BFF1–3) and two pyranose forms (AFP1–2 and BFP1–2) were selected to represent the α and β anomers of fructose, as detailed in the Computational Details section. Due to the inherent limitations of AIMD simulations, the results indicate that the configuration space explored is incomplete for both the furanose and pyranose cases. Figures S1–S4 display 2D probability distributions of dihedral angles, which are useful for characterizing conformational space and flexibility. These figures highlight the regions of configuration space explored by classical and ab initio MD simulations. The chosen dihedrals are associated with hydroxymethyl groups in the investigated systems and OH groups linked to C2 in pyranose structures.

**Table 1:** The Probability of Occurrences of Dihedral Angles for the Studied Fructose Systems Obtained from Classical and Ab Initio MD Simulations^a^

				Ab initio MD								
	Class. MD			AFF1			AFF2			AFF3		
AFF	*g* ^–^	*g* ^**+**^	*t*	*g* ^–^	*g* ^**+**^	*t*	*g* ^–^	*g* ^+^	*t*	*g* ^–^	***g** ^**+**^*	***t***
C2–C1–O1–H1	0.34	0.64	0.02	0.00	0.45	0.55	0.53	0.43	0.04	0.98	0.00	0.02
C5–C6–O6–H6	0.32	0.52	0.16	1.00	0.00	0.00	0.15	0.13	0.72	0.98	0.00	0.02
O5–C2–C1–O1	0.84	0.13	0.03	1.00	0.00	0.00	0.00	0.00	1.00	0.00	0.00	1.00
O5–C5–C6–O6	0.59	0.40	0.01	0.00	1.00	0.00	0.00	1.00	0.00	0.00	1.00	0.00

				Ab initio MD					
	Class. MD			AFP1			AFP2		
AFP	*g* ^–^	*g* ^**+**^	*t*	*g* ^–^	*g* ^**+**^	*t*	*g* ^–^	*g* ^+^	*t*
C2–C1–O1–H1	0.44	0.44	0.12	0.00	0.91	0.09	0.28	0.60	0.12
O6–C2–O2–H2	0.45	0.50	0.05	1.0	0.00	0.00	1.00	0.00	0.00
O6–C2–C1–O1	0.12	0.88	0.00	0.00	1.00	0.00	1.00	0.00	0.00

				Ab initio MD								
	Class. MD			BFF1			BFF2			BFF3		
BFF	*g* ^–^	*g* ^**+**^	*t*	*g* ^–^	*g* ^**+**^	*t*	*g* ^–^	*g* ^+^	*t*	*g* ^–^	***g** ^**+**^*	***t***
C2–C1–O1–H1	0.45	0.54	0.00	1.00	0.00	0.00	0.00	1.00	0.00	0.00	0.55	0.45
C5–C6–O6–H6	0.38	0.52	0.10	0.88	0.00	0.12	0.00	0.85	0.15	0.00	1.00	0.00
O5–C2–C1–O1	0.30	0.50	0.20	1.00	0.00	0.00	1.00	0.00	0.00	1.00	0.00	0.00
O5–C5–C6–O6	0.43	0.57	0.00	0.00	0.00	1.00	1.00	0.00	0.00	1.00	0.00	0.00

				Ab initio MD					
	Class. MD			BFP1			BFP2		
BFP	*g* ^–^	*g* ^**+**^	*t*	*g* ^–^	*g* ^**+**^	*t*	*g* ^–^	*g* ^+^	*t*
C2–C1–O1–H1	0.27	0.57	0.16	0.00	0.00	1.00	0.00	1.00	0.00
O6–C2–O2–H2	0.34	0.00	0.66	0.72	0.28	0.00	1.00	0.00	0.00
O6–C2–C1–O1	1.00	0.00	0.00	1.00	0.00	0.00	1.00	0.00	0.00

a AFF: α-d-fructofuranose, BFF: β-d-fructofuranose, AFP: α-d-fructopyranose, BFP: β-d-fructopyranose.

Compared with the pyranose form of fructose, the furanose ring is known to be significantly more flexible,^9^ thanks to the small free-energy barriers between its different ring conformations. We found, in agreement with previous work,^9^ that in both ab initio and classical MD simulations, the α- and β-d-fructopyranose molecules adopt ^5^C_2_ and ^2^C_5_ ring conformations, respectively. On the other hand, the anomers of the furanose ring exhibit a diverse conformational landscape (puckering). Figure 3 presents the probability distributions of puckering states in our classical and ab initio MD simulations. The entire Cremer-Pople pseudorotation wheel, including the pseudorotational angles, is illustrated in Figue S5.

**Figure 3:**
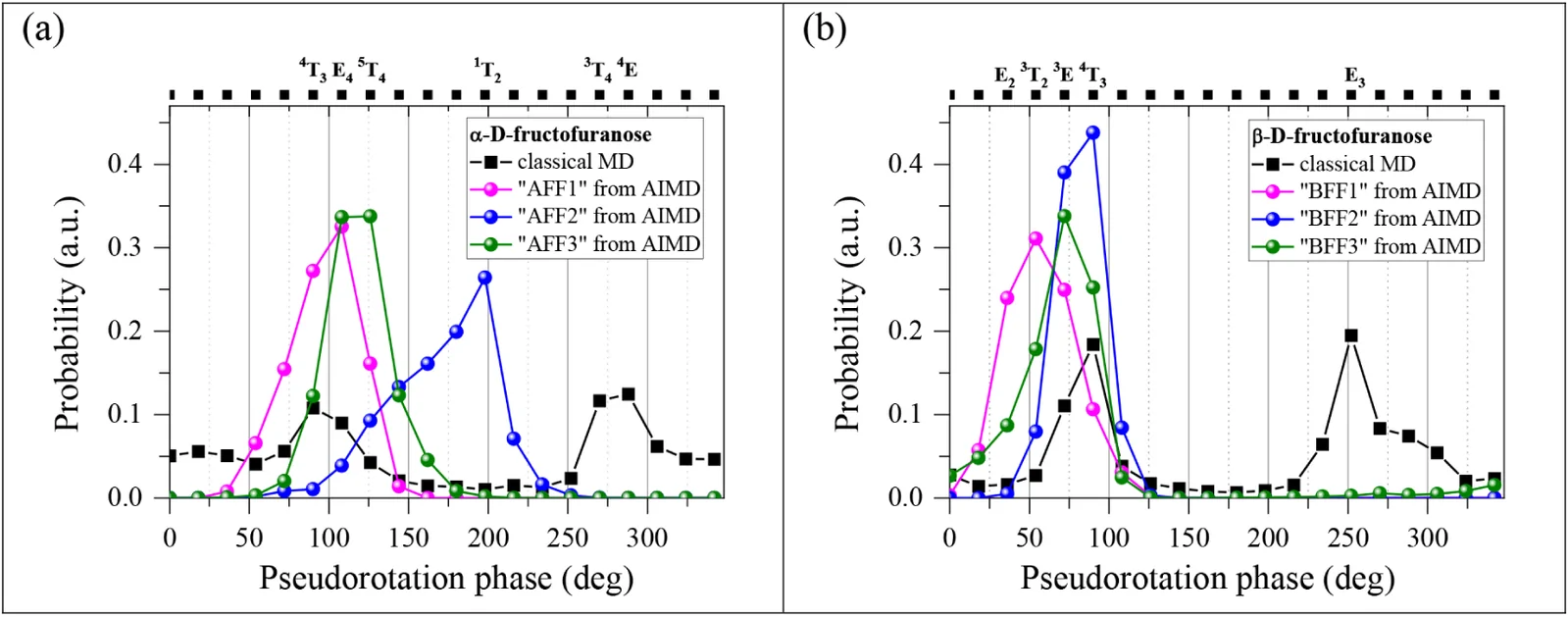
Probability functions of the different puckering states calculated from classical MD and from ab initio MD for (a) α-d-fructofuranose and (b) β-d-fructofuranose.

Concerning classical MD models, both α- and β-fructofuranose exhibit two distinct peaks. One of the peaks corresponds to the ^4^T_3_ geometry for both systems. The ^4^T_3_ twist conformation provides high structural stability for β-d-fructofuranose by maximizing the spatial separation of the large exocyclic groups. In contrast, for the α-anomer, this geometry forces the C1 and C6 hydroxymethyl branches into a more crowded spatial arrangement on the same face of the ring. Inverting the ring puckering to the ^3^T_4_ conformation, the large exocyclic groups of the α-anomer achieve optimal spatial separation on opposite faces of the ring. This ^3^T_4_ twist structure, together with the ^4^E envelope geometry, composes the second peak for α-d-fructofuranose, while the second peak of β-d-fructofuranose corresponds to the E_3_ position. Notably, in both the ^4^E and E_3_ envelope geometries, the C6 hydroxymethyl and C1 branches are oriented toward the exposed, outer face of the ring, thereby enhancing the solvent accessibility of these groups. Low but noticeable intensities are observed at angles below the first peak and above the second peak.

Higher occurrence of the pseudorotation phase angle arises from AIMD simulations, but it is limited to smaller ranges. The maximum values are for the three α-d-fructofuranose models: 108° (E_4_), 108°–126° (E_4_,^5^T_4_) and 198° (^1^T_2_). For β-d-fructofuranose, the maximum values are 54° (^3^T_2_), 72° (^3^E), and 90° (^4^T_3_). Here, we note that the shapes of these distributions closely resemble those of Gaweda et al.^28^

To determine if the hydroxymethyl groups exhibit any preference in the more favored puckering cases, we calculated the pseudorotation angle as a function of the dihedral angles of the hydroxymethyl groups of furanose rings from the classical MD simulation. The results are shown in Figure 4 for O5–C5–C6–O6 and O5–C2–C1–O1 dihedrals. The 2D maps for C5–C6–O6–H6 and C2–C1–O1–H1 dihedrals are placed in Figure S6.

**Figure 4:**
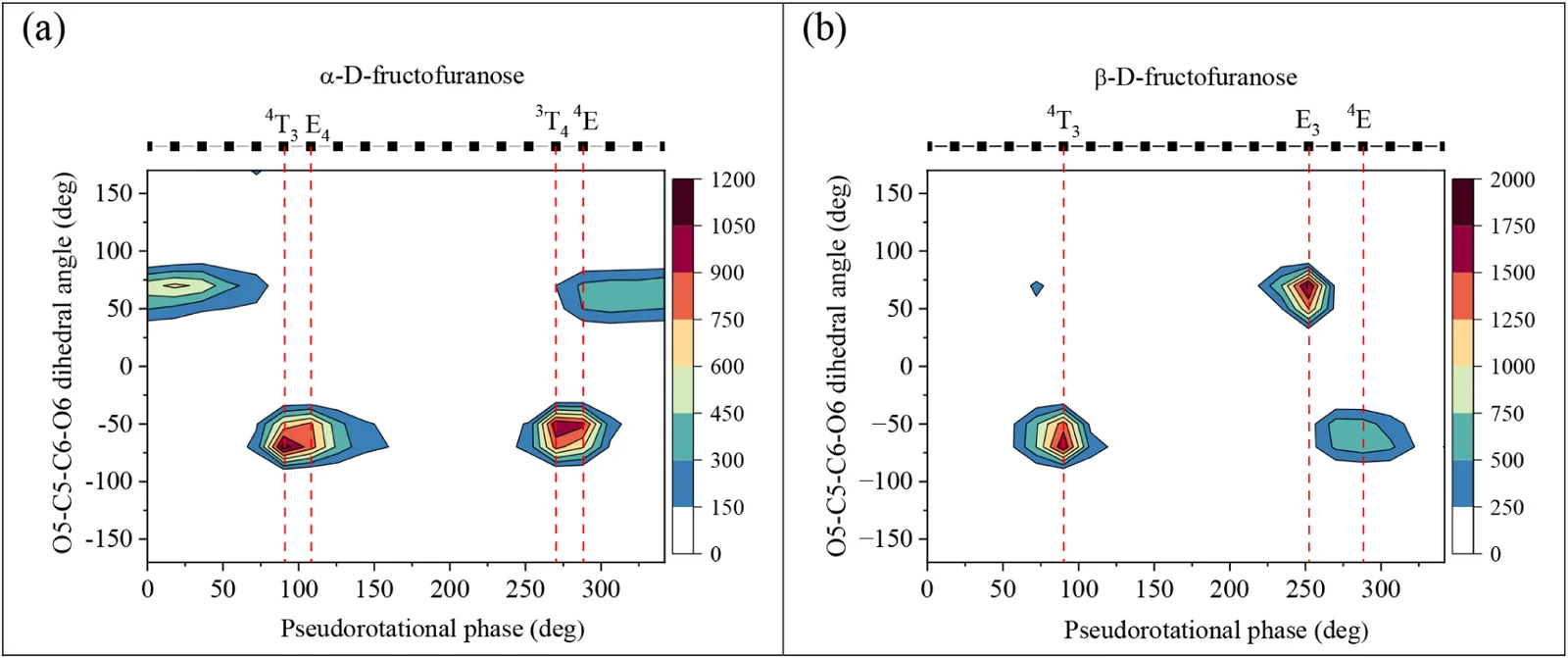
2D density distribution functions of the pseudorotation phase angle and dihedral angles connecting to the hydroxymethyl group calculated from classical MD for (a) α-d-fructofuranose and (b) β-d-fructofuranose.

Concerning α-d-fructofuranose, the 2D density distribution of the O5–C5–C6–O6 dihedral angle shows a significant *g* ^–^ preference, where the most abundant puckering cases (^4^T_3_, E_4_, ^3^T_4_, and ^4^E) were observed. The ^4^T_3_ and ^4^E geometries effectively minimize the distance between the two hydroxymethyl groups, facilitating exocyclic (OH1)–exocyclic (OH6) hydrogen bonds. In contrast, the E_4_ conformation brings the exocyclic C6 group into proximity with the ring oxygen. Furthermore, the ^3^T_4_ state promotes linkage between the OH1 or OH6 and OH3 group.

For β-d-fructofuranose, the conformational distribution reveals a clear coupling between ring puckering and exocyclic chain rotation, defining two distinct structural states. The *g* ^+^ preference for E_3_ represents an “open” conformation, thus maximizing its exposure to the solvent. The OH6 exocyclic group becomes closer to the center of the ring, allowing it to easily form hydrogen bonds with either the ring oxygen (O5) or the anomeric region (OH1/OH2). Conversely, the *g* ^–^ preference for the ^4^T_3_ ring stabilizes a “closed” conformation, which is a sterically crowded environment on the top face of the sugar, resulting in a dense network of intramolecular hydrogen bonds associated with the OH6 and O5 interaction. In summary, the orientation of the hydroxymethyl group is strongly coupled to the preferred puckering conformation, which directly shapes the hydration properties of the fructose systems.

### Intramolecular H-Bond Properties

To identify H-bond connections, we employ a geometric definition based on the distance between oxygen and hydrogen atoms, as well as the O–H···O angle. The distinct first intermolecular minimum of the O–H radial distribution function, located at 2.5 Å, determined the limiting distance. These functions for the water subsystems are shown in Figure S7.

In the case of the O–H···O angle, usually the applied limit is 30°. For the intermolecular case, this definition is also acceptable in our cases (c.f. Figure S8). However, intramolecular angular distribution functions for OH groups (Figure 5) indicate that measurable intensity within 30° is present only for α-d-fructopyranose. Additionally, applying this strict definition for β-d-fructopyranose, the intramolecular H-bond cannot be detected. Note that Figure 5 summarizes the results for each OH group, but the angle distributions do not detail all possible O–H···O pairs individually.

**Figure 5:**
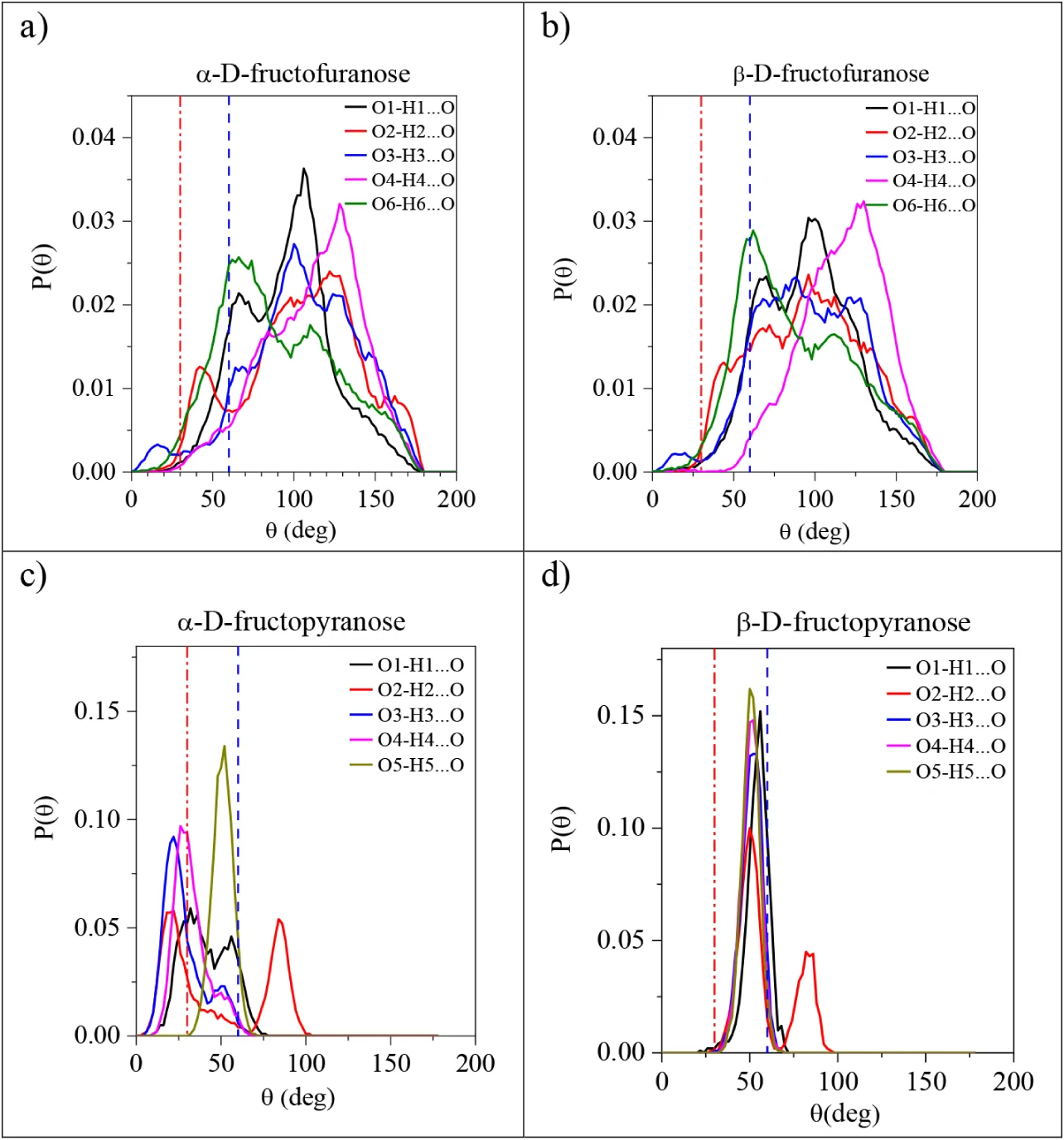
Angular distributions of intramolecular O–H···O angles (θ) based on classical MD simulations. (a) α-d-fructofuranose, (b) β-d-fructofuranose, (c) α-d-fructopyranose and (d) β-d-fructopyranose. The 60° threshold is marked in blue, and the 30° threshold is marked in red.

To calculate the probabilities of intramolecular H-bonds, a less strict angle definition of O–H···O < 60° was used. Figure 6 shows the probability of each OH group or ring oxygen being bonded within the molecule to another OH group or ring oxygen. The results with the stricter definition (O–H···O < 30°) are presented in Table S1. Only classical MD simulation models were used for the calculations presented in Figures 5–6, because the orientation time of OH groups in AIMD simulations is approximately 100 ps. This time period does not allow fructose molecules to leave their initial state.

**Figure 6:**
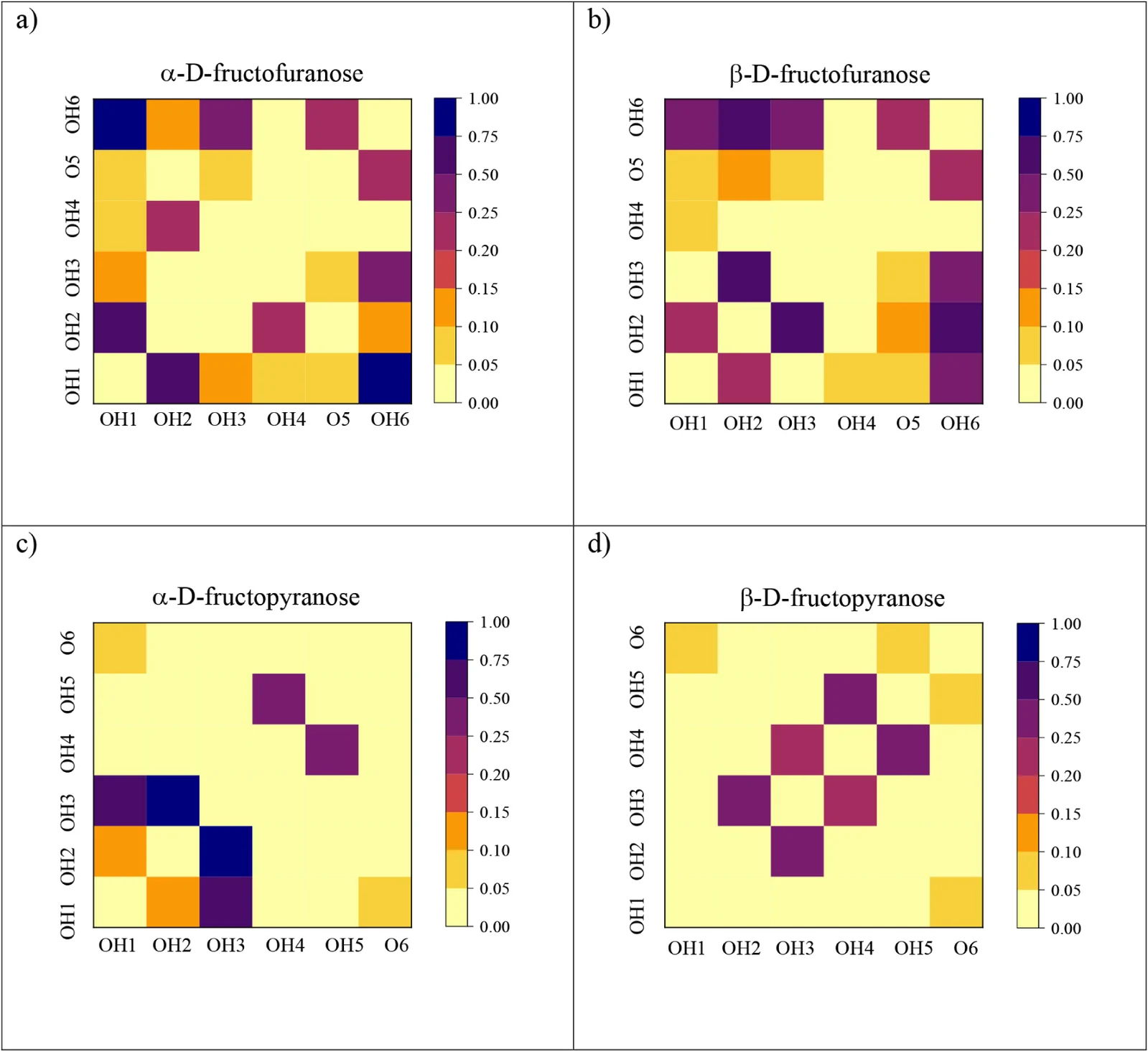
Intramolecular H-bond probabilities calculated from classical MD models based on the less strict definition (O–H···O < 60° and r­(O···H) < 2.5 Å). (a) α-d-fructofuranose, (b) β-d-fructofuranose, (c) α-d-fructopyranose and (d) β-d-fructopyranose.

Figure 6 confirms that the formation of H-bonds within the molecule depends significantly on both the molecule’s conformation (ring puckering state) and the configuration of the OH groups. In the two fructofuranose systems, the two hydroxymethyl groups (OH1 and OH6), along with the interaction between OH6 and ring oxygen (O5), are primarily responsible for forming H-bonds within the molecule, which is fully consistent with our findings from Figure 4. Additional pronounced intramolecular H-bonding interactions were identified between the OH6 and OH3, OH2 and OH4, as well as OH2 and OH1 groups. Note that these observations directly align with the molecular dynamics findings of Buğday et al.^18^ These specific linkages demonstrate that the hydrogen-bonding network extends beyond the primary exocyclic-exocyclic pairs, involving both the anomeric region and the ring hydroxyls.

On the other hand, for β-d-fructofuranose, a higher probability of H-bonding was observed between the OH2 and OH6 groups, which, in light of our earlier conclusions, is fully consistent with the E_3_/*g* ^+^ conformational state. Additionally, the remarkably high probabilities of the OH1-OH2, OH2-OH3, and OH3-OH6 interactions are also linked to this conformational state. It is important to note that this does not imply the simultaneous presence of all three hydrogen bonds; rather, this single conformation provides the most flexible platform that geometrically accommodates the dynamic fluctuations among these interactions.

In fructopyranose systems, the number of OH groups participating in H-bonds is less than in the fructofuranose case; moreover, the probability of occurrence is lower. This can be explained by the significantly lower flexibility of the pyranose ring. Furthermore, the ring oxygen is not involved in the H-bond within the molecule. In the case of α-d-fructopyranose, we can conclude that OH groups that are closer to each other can form H-bonds; this means that the OH1–OH3, OH2–OH3, and OH4–OH5 pairs can interact. In contrast, the OH3-OH4 connection is missing because these hydroxyl groups point in opposite directions within the chair conformation.

Between them, the highest probability was found between the OH2 and OH3 groups. It is important to note that when applying a stricter definition of H-bond (O–H···O < 30°), H-bonds cannot be found in β-d-fructopyranose; even with a less strict definition (O–H···O < 60°), we still observe the lowest values among the four systems studied. Hydrogen bond interactions can be identified only for the OH2–OH3, OH3–OH4, and OH4–OH5 pairs. The OH1–OH2 interaction is missing due to the structural constraints of the ^2^C_5_ chair. The axial-equatorial split between the OH2 group and the C1 arm prevents these neighboring groups from establishing a proper H-bonding geometry. Interestingly, the H-bond between the OH3 and OH4 groups in the β anomer is enabled by both hydroxyl groups occupying vicinal equatorial positions, which brings them into close spatial proximity. Conversely, in α-d-fructopyranose, these groups are forced into an axial-equatorial split, causing them to point away from each other and preventing the establishment of a stable H-bond.

### Intermolecular H-Bond Properties

The average coordination numbers regarding the first hydration shell are in Table 2. The hydroxyl groups of sugar molecules prefer to form H-bonds with surrounding water molecules (HPHILE). On the other hand, the C–H parts of the sugar molecule have a limited ability to interact with water. However, the water molecules can still find themselves in the well-defined (close) environment of the sugar molecule thanks to weak hydrophobic interactions, such as van der Waals forces and CH···O interactions with water, even in the absence of H-bonding. Water molecules for which the condition r­(C_i_···O_water_) < 4.5 Å is satisfied are considered members of the hydrophobic shell (HPHOBE). The accuracy of these calculations was found to be within 0.05 using the bootstrapping method^29,30^ for both the hydrophilic and hydrophobic hydration cases. It is worth noting that we also tested the robustness of this criterion in the 4.5–5 Å range, and the results showed that changes to the criterion do not affect the main conclusions.

**Table 2:** Average Coordination Numbers for the Studied Fructose Systems

	Ab initio MD						Classical MD	
	AFF1	AFF2	AFF3	BFF1	BFF2	BFF3	AFF	BFF
HPHILE	10.1 ± 1.4	10.9 ± 1.3	11.2 ± 1.6	8.8 ± 1.4	10.9 ± 1.4	10.2 ± 1.6	7.5 ± 1.8	7.7 ± 1.9
HPHOBE	9.8 ± 1.3	9.3 ± 1.4	9.8 ± 1.5	11.7 ± 1.7	8.2 ± 1.5	10.5 ± 1.6	11.8 ± 2.0	12.6 ± 2.1

	Ab initio MD				Classical MD	
	AFP1	AFP2	BFP1	BFP2	AFP	BFP
HPHILE	6. 5 ± 1.3	8.0 ± 1.3	9.7 ± 1.7	9.4 ± 1.6	6.4 ± 1.7	7.7 ± 1.7
HPHOBE	14.5 ± 1.9	11.7 ± 1.8	12.2 ± 2.1	12.4 ± 2.0	12.1 ± 2.3	11.7 ± 2.4

In general, H-bonded (HPHILE) and non-H-bonded (HPHOBE) water molecules are present in comparable numbers in the vicinity of a fructose molecule. No clear trend is observed in the number of H-bonds between the α- and β-anomers of fructofuranose molecules, owing to the positions of the two flexible hydroxymethyl groups, which significantly influence the local water environment. On the other hand, in fructopyranose systems, the β-fructopyranose forms more hydrogen bonds with water than the α form. The difference is more pronounced in the AIMD simulation.

The H-bonding ability of the different oxygen sites (for the assignment, c.f. Figure 1) is shown in Figure 7. Figure 7a–c contain the results from classical MD simulations for all four studied fructose systems. The outcomes from ab initio calculations for the four fructopyranose models are presented in Figure 7d–f. Fructofuranose systems lack clear trends, as previously discussed, so we will not examine them here; results can be found in Figure S9. Fructose molecules contain five hydroxyl groups, each capable of donating one hydrogen bond and accepting two hydrogen bonds. Additionally, the ring O atom can form two H-bonds with water, as an acceptor. Figure 7a and Figure 7d present how many H-bonds each O or OH site makes. Figures 7b and 7e show the extent to which it participates as a donor, and Figures 7c and 7f contain the extent to which it participates as an acceptor.

**Figure 7:**
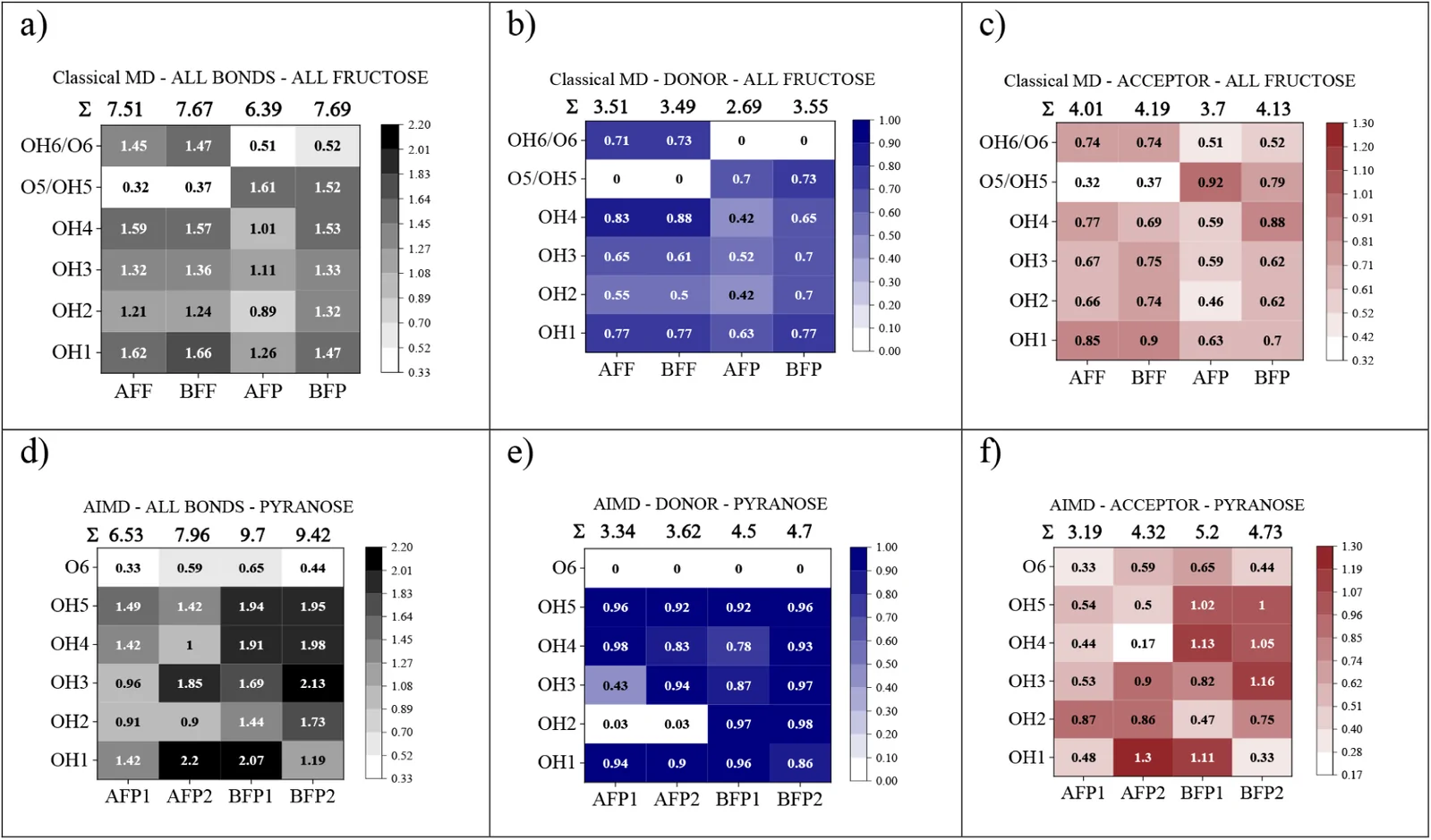
Average number of H-bonds together with the acceptor and donor H-bond numbers. a) All H-bonds for all fructose systems from classical MD, b) donor type H-bonds for all fructose systems from classical MD, c) acceptor type H-bonds for all fructose systems from classical MD, d) all H-bonds for fructopyranose systems from AIMD, e) donor type H-bonds for fructopyranose systems from AIMD, and f) acceptor type H-bonds for fructopyranose systems from AIMD.

Classical MD simulations indicate that the number of H-bonds for the β-anomers typically exceeds that for the α-anomers. For the furanose systems, this difference arises from the acceptor capacity, whereas their donor counts remain nearly identical. In contrast, the β-pyranose anomer shows higher numbers in both donors and acceptors. With a good approximation, almost half of the hydrogen bonds are formed as acceptors, while the other half are formed as donors. Interestingly, despite the greater potential for oxygen atoms to act as acceptors, this capacity is not fully utilized to form more H-bonds. Concerning donor properties, the OH4 sites are the best donors for furanose systems. In the case of pyranose, the OH5 site for α-d-fructopyranose and the OH1 site for β-d-fructopyranose are the most occupied. In addition to this latter case, the hydroxymethyl groups (OH1 and OH6 in furanose, as well as OH1 in pyranose systems) were found to be significant in the other three systems. The best acceptor is the OH1 hydroxymethyl group for furanose systems. The two pyranose systems show slightly different behaviors as acceptors; the OH5 site was found to be the most attractive for α-d-fructopyranose, and the OH4 site for β-d-fructopyranose. Note that for the β-anomer, the OH5 site also represents the second most attractive site for water interaction. These observations regarding β-d-fructopyranose directly align with the molecular dynamics findings of Buğday et al.,^18^ but the α-pyranose tautomer was not investigated in their study. Except at this specific site (OH5), the β-anomer exhibits higher donor and acceptor counts, resulting in a larger overall H-bonding capacity. Interestingly, while the α-anomer shows more localized preferences, the interaction sites on the β-pyranose are characterized by a much more uniform efficiency across the molecule. This enhanced propensity for H-bonding with the solvent may well explain the higher solubility typically observed for the β-anomer. In each case that was studied, the ring oxygens were found to be the least effective acceptors.

Overall, the main qualitative trends in hydrogen-bonding patterns are very similar in both classical and ab initio MD simulations. However, a few quantitative differences are observable between the two methods, most notably at the OH2 (donor) and OH4 (acceptor) sites of α-d-fructopyranose, where AIMD yields significantly lower intermolecular H-bond numbers. This quantitative difference originates from the persistent intramolecular H-bonding network captured by AIMD at these specific sites (c.f. Figure 6c), which reduces their availability for intermolecular hydration. These differences between the two computational approaches arise from how they treat electronic interactions and sampling: while classical force fields use fixed partial charges and may overestimate water competition, AIMD explicitly accounts for electronic polarization and dynamic charge transfer, which stabilizes specific internal H-bonds. Additionally, the shorter trajectory length of AIMD emphasizes these persistent conformational states, whereas classical MD samples a broader equilibrium ensemble over nanosecond time scales.

Water molecules were classified based on the number of H-bonds they take part in as H-donors (n_D_ = 0, 1, or 2) and H-acceptors (n_A_ = 0, 1, 2, or 3). The 0D:0A case indicates that water molecules are in a no-bond situation (or state), while 2D:3A shows that the water molecule is fully bonded. The occurrences of possible bonding states are shown in Table 3. Almost two-thirds of the water molecules are four-coordinated; more specifically, they are in a 2D:2A state. Another about 25% are three-coordinated. Significantly more molecules are in the 2D:1A than the 1D:2A state. The 1D:1A and 2D:3A cases are less relevant. Comparing the data of water molecules bound to fructose (1^*st*^) with those surrounded exclusively by other water molecules (*no 1* ^*st*^) indicates that the H-bonding behavior of water molecules is almost independent of whether they are directly associated with the fructose molecule. In furanose cases, the values depend largely on the model, i.e., the local positions of the hydroxyl groups originally given, because the simulation time is shorter than the interconversion between conformers.

**Table 3:** Fractions of Water Molecules as H-Acceptors and as H-Donors in the H-Bonds^a^

	1D:1A		1D:2A		2D:1A		2D:2A		2D:3A	
	1^st^	no 1^st^	1^st^	no 1^st^	1^st^	no 1^st^	1^st^	no 1^st^	1^st^	no 1^st^
AFF1	0.055	0.056	0.103	0.103	0.147	0.151	0.637	0.630	0.039	0.037
AFF2	0.048	0.072	0.072	0.114	0.193	0.165	0.575	0.581	0.035	0.040
AFF3	0.037	0.037	0.074	0.062	0.163	0.150	0.652	0.681	0.020	0.017
BFF1	0.038	0.067	0.062	0.114	0.161	0.156	0.648	0.603	0.032	0.039
BFF2	0.034	0.060	0.069	0.100	0.169	0.152	0.602	0.629	0.052	0.039
BFF3	0.049	0.065	0.078	0.110	0.175	0.160	0.611	0.608	0.031	0.035
AFP1	0.033	0.070	0.087	0.122	0.160	0.161	0.659	0.589	0.042	0.035
AFP2	0.035	0.046	0.067	0.095	0.145	0.131	0.613	0.690	0.050	0.027
BFP1	0.037	0.066	0.089	0.108	0.131	0.154	0.646	0.615	0.043	0.035
BFP2	0.038	0.067	0.092	0.110	0.150	0.155	0.610	0.607	0.037	0.039

a *1^st^: water molecules bound to fructose; no 1^st^*: water molecules in a bulk water environment (surrounded only by other water molecules).

### Electronic Properties of Fructose

Based on AIMD simulations, the electronic properties of the fructofuranose and fructopyranose molecules were characterized using Bader charges (Figure 8).^31^ Additionally, atomic dipoles corresponding to different O, C, and OH sites can be found in Figure S10. These properties depend on both intra- and intermolecular interactions. In all systems, the smallest negative Bader charge is located at the ring oxygen atoms (O5 for furanoses and O6 for pyranoses). Due to this low electron density, the ring oxygen acts as the weakest H-bond acceptor within the molecule. Consequently, it is less attractive to the hydrogen atoms of water molecules than the electron-rich oxygens of the hydroxyl (−OH) groups, thereby explaining why it forms so few hydrogen bonds with water. The charge of the C atoms forming the ring is the smallest at the C2 position. This unique behavior of the C2 position can be attributed to its role as the anomeric center in fructose. Except for this C2, the values of the other ring carbon atoms are of similar magnitude. Interestingly, the charges of the hydroxymethyl carbon atoms (C1 and C6 for furanoses, and C1 for pyranoses) fall within the same range.

**Figure 8:**
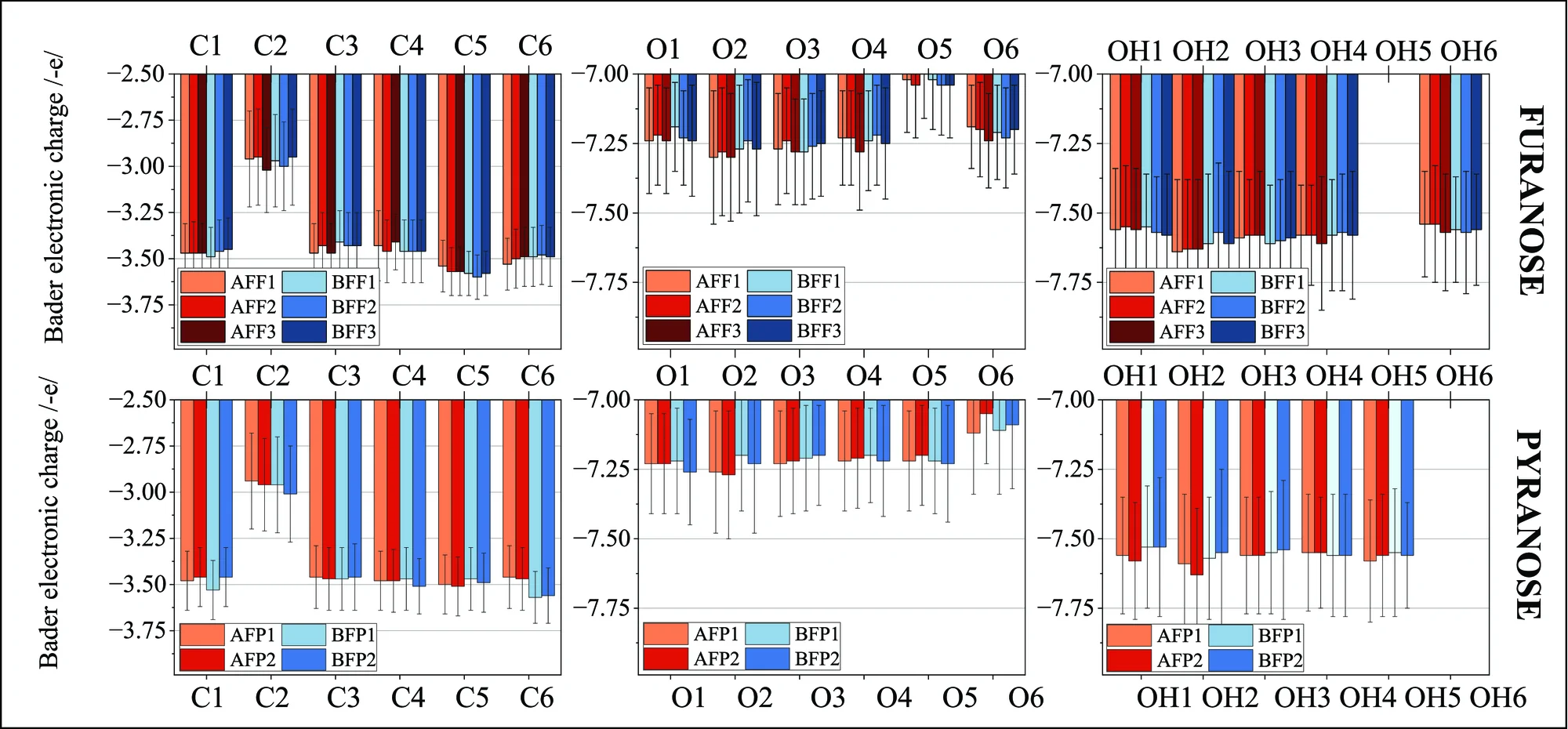
Bader charges /–e/on the C, O, and OH sites of the fructose molecule in the solutions based on AIMD simulations. (Note: the C has a different y scale from the others).

Regarding the OH sites, the charge at OH2 is slightly larger for the α-anomers. This trend is observed for both furanose and pyranose rings and originates from the stereoelectronic configuration of the anomeric center (C2). In the α-anomers, the spatial orientation of the exocyclic hydroxyl group (OH2) relative to the ring oxygen minimizes steric hindrance and optimizes hyperconjugation (the anomeric effect). This specific conformation allows the oxygen of the OH2 group to retain a slightly higher electron density—resulting in a larger negative Bader charge—compared to the more sterically strained or electronically altered environment of the β-anomers. It is important to point out that these calculations exhibit significant fluctuations.

The examination of the dipole moment values reveals similar conclusions: only two somewhat outstanding values were found, with notable deviations occurring only at the C2 (higher) and ring oxygen (lower) positions, as illustrated in Figure S10.

### Electronic Properties of Water

The Wannier dipole moment of water molecules directly hydrogen-bonded to a fructose molecule was examined and plotted in Figure 9. As we previously demonstrated,^32^ these dipole moments depend on the water molecule’s environment, specifically the number of H-bonded neighbors. These neighbors are, on the one hand, the fructose molecule itself, and, on the other hand, the other water molecules surrounding the water molecule. Identifying a clear trend in the dipole moments of individual OH groups and oxygens is very challenging due to the inherent flexibility of the fructose molecule, namely, free OH rotation and various ring puckering states. While our setup includes multiple models for each anomer (three for each furanose and two for each pyranose form), they represent distinct local conformations that do not span the complete configurational space. As a result, the dipole moments are highly sensitive to the specific local environment of each model, rather than exhibiting a generalized pattern. Therefore, we averaged the data separately for each of the four main fructose systems, as presented in Figure 6. The calculated values for all 10 AIMD models are plotted in Figures S11–S12. In general terms, the water molecules surrounding the fructose molecule are primarily fourfold coordinated, connecting to one fructose molecule and three water molecules as neighbors. This statement holds for both pyranose and furanose forms, regardless of whether the fructose molecule acts as a donor or an acceptor, and regardless of the oxygen atom to which it is bonded. (c.f. Table 3) The dipole moment of fourfold-coordinated water molecules is larger than that of threefold-coordinated ones. This is consistent with findings related to pure water^32^ and other pyranose monosaccharides.^23^ The results indicate that, in almost all cases, when the sugar molecule acts as a hydrogen donor, the dipole moment of the water molecules is generally larger than when it acts as an acceptor. This is in line with the experimental fact that the OH groups of sugars are more acidic than the OH group of water.^33–35^ Water molecules can only form H-bonds as donors with ring oxygen atoms (O5 for furanose and O6 for pyranose ring). However, the dipole moments of these water molecules do not differ significantly from those of other OH groups when the sugar molecule also acts as an H-bond acceptor.

**Figure 9:**
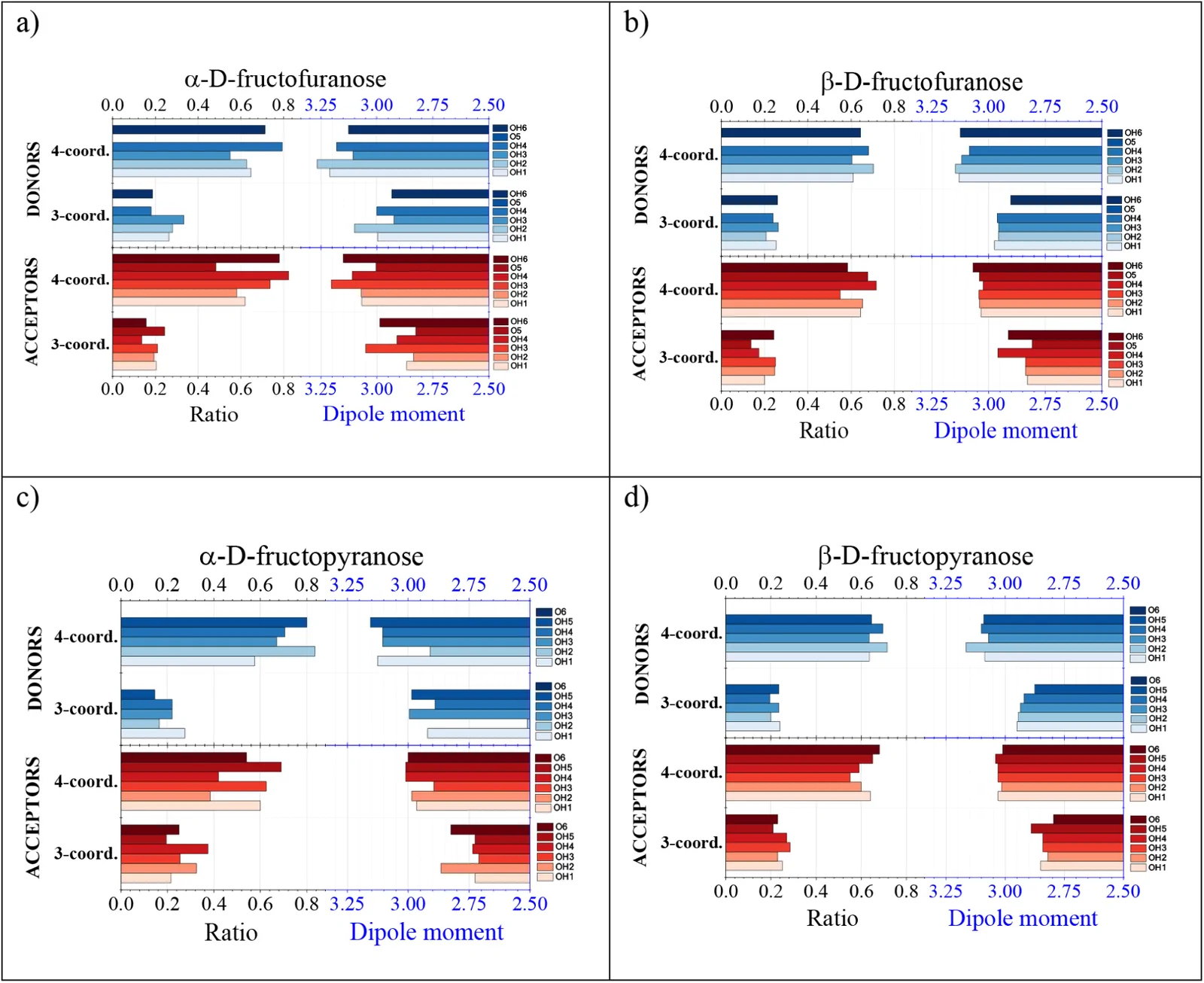
Properties of three- and four-coordinated water molecules around the fructose molecule based on AIMD simulations. (Note that the acceptor or donor nature refers to the fructose molecules.) (a) α-d-fructofuranose, (b) β-d-fructofuranose, (c) α-d-fructopyranose and (d) β-d-fructopyranose. Note that the dipole moment was calculated by the Wannier approach.

Table 4 lists the Wannier dipole moments of water molecules that are not directly bonded to the sugar molecule. Additionally, the values are provided separately based on the bonding condition of the water molecules. Note that the probabilities of occurrence of these bonded states (1D:1A, 1D:2A, 2D:1A, 2D:2A, and 2D:3A) are shown in Table 3. In each system, the dipole moment of water molecules, as previously established,^23^ depends on the number of H-bonded neighbors. Furthermore, a significant difference can be observed between water molecules in a 1A:2D environment and those in a 2A:1D H-bonding environment, despite both having the same number of H-bond connections (threefold-coordinated). This result indicates that the dipole moment of water molecules is influenced not only by the quantity, but also by the types of H-bonds formed, specifically the number of acceptor type and donor type H-bonds. On the other hand, no significant difference in these dipole moment values can be observed between molecules in the first H-bonding sphere and those further away. From this perspective, systems with five- and six-membered rings behave similarly.

**Table 4:** Dipole Moment of Water Molecules Based on AIMD Simulation, Classified by the Bonded State

	1D:1A		1D:2A		2D:1A		2D:2A		2D:3A	
	1^st^	no 1^st^	1^st^	no 1^st^	1^st^	no 1^st^	1^st^	no 1^st^	1^st^	no 1^st^
AFF1	2.81	2.77	3.03	3.01	2.94	2.92	3.20	3.18	3.19	3.24
AFF2	2.83	2.77	2.99	2.98	2.91	2.89	3.15	3.12	3.20	3.15
AFF3	2.86	2.80	3.05	3.02	2.95	2.96	3.16	3.20	3.23	3.25
BFF1	2.72	2.72	2.95	2.93	2.89	2.86	3.09	3.08	3.15	3.13
BFF2	2.74	2.72	2.94	2.93	2.89	2.86	3.1	3.09	3.17	3.12
BFF3	2.74	2.7	2.89	2.91	2.86	2.85	3.07	3.07	3.08	3.1
AFP1	2.71	2.71	2.91	2.91	2.85	2.85	3.06	3.06	3.1	3.1
AFP2	2.71	2.73	2.94	2.93	2.84	2.88	3.1	3.1	3.13	3.14
BFP1	2.75	2.71	2.92	2.91	2.84	2.85	3.07	3.07	3.11	3.11
BFP2	2.72	2.7	2.93	2.91	2.85	2.85	3.07	3.07	3.13	3.11

### Hydration Sphere Properties

We can obtain a more detailed picture of the arrangement of water molecules around fructose in the aqueous phase by calculating the spatial density function in a fructose-fixed local coordinate system. The most likely locations where H-bonds form between sugar and water molecules can be observed in the direction of the electron pairs of the O atoms and in the direction of the OH bond of the sugar molecule in each case. It should be noted here that the significant water occurrence around the ring O atom is not due to the ring O but to the OH1, OH2, and OH6 groups for furanose cases, as well as the OH1 and OH2 for pyranose cases. Water molecules that do not form H-bonds are more likely to occur in directions determined by CH2 or CH bonds. Figure 10 provides quantitative information about the water molecule’s arrangements around the fructose molecule. A coordinate system was defined with the center at the center of mass (CM) of the sugar molecule. The XY plane is defined by the positions of C5 and C2 atoms for fructofuranose, as well as C6 and C2 atoms for fructopyranose, and the x direction is defined as the bisector of the angle of C5–CM–C2 and C6–CM–C2, respectively. The positive direction points to O5 (furanose) or O6 (pyranose). Using this coordinate system, we can determine the number of molecules located above (z > 0) and below (z < 0) the plane defined by the sugar molecule, and within the regions defined by the inequalities x > 0, x < 0, and y > 0, y < 0. In this specific coordinate system, the local water density depends on both the ring geometry (which is substantially more flexible in fructofuranose forms) and, most importantly, on the conformation of the hydroxyl and hydroxymethyl groups. A schematic representation of the coordinate system used is provided in Figure S13.

**Figure 10:**
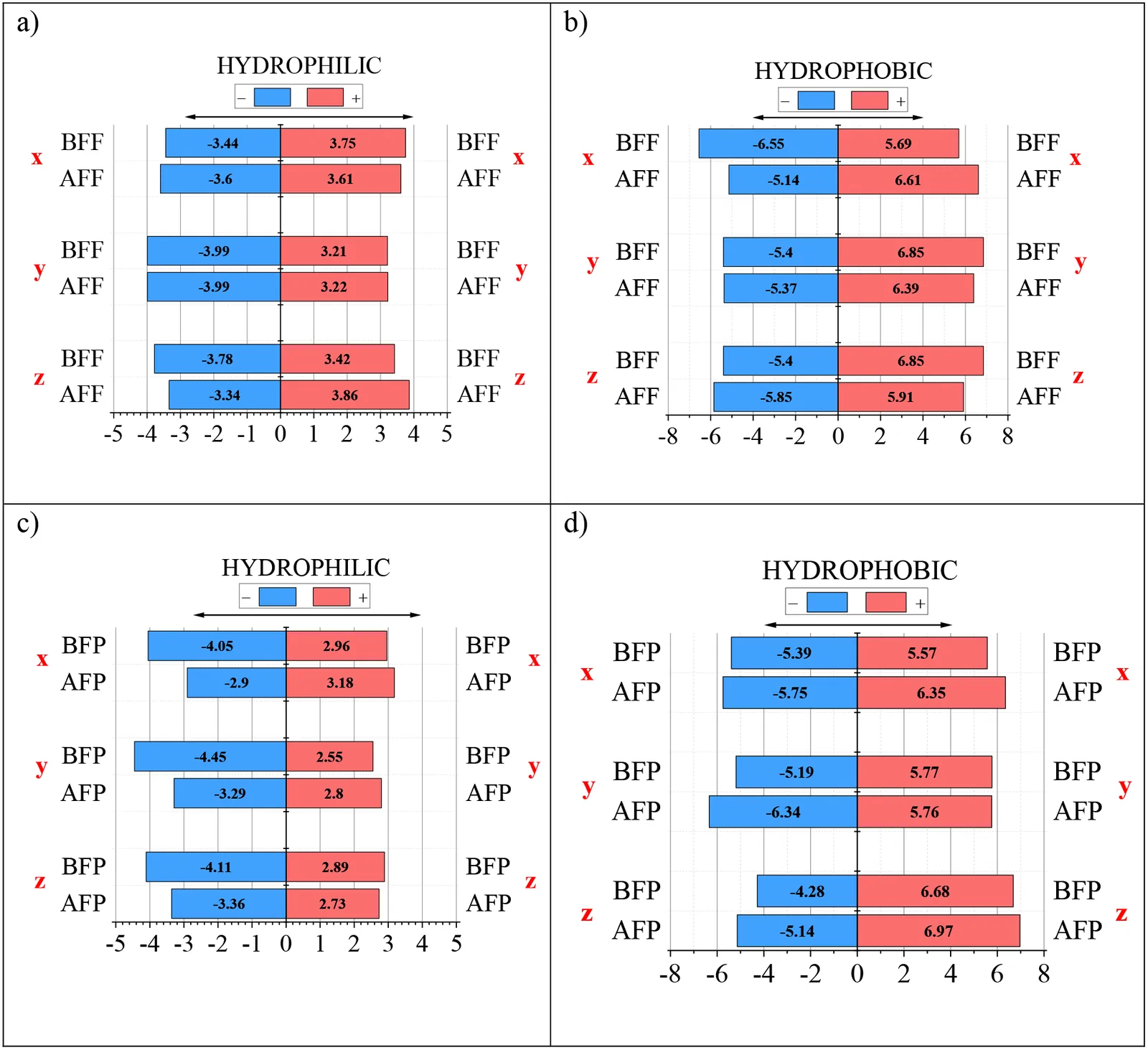
Number of hydrophobic and hydrophilic H-bonded water molecules above and below the XY, YZ, and XZ planes of fructose molecules based on classical MD simulations. a) hydrophilic contribution for fructofuranose systems, b) hydrophobic contribution for fructofuranose systems, c) hydrophilic contribution for fructopyranose systems, and d) hydrophobic contribution for fructopyranose systems.

The results from classical MD simulations are presented in Figure 10, while those from AIMD simulations—which follow a similar trend—are shown in Figure S14. The shape of the hydrophilic shell of furanose systems is very similar in the x and y directions. The difference between the two fructofuranose conformers is most pronounced in the *z*-direction, which can be rationalized by the specific spatial orientation of their two hydroxymethyl groups along this axis. The opposing *x*-directional shifts of the hydrophobic character between the two anomers can be explained by the stereochemical reconfiguration at the anomeric center (C2).

The hydrophilic sphere of β-d-fructopyranose is more asymmetrical than that of the α anomer, with higher water density around the −x, −y, −z region. This can be explained by the fact that this domain represents the most sterically open and least hindered side of the ring, which is made highly attractive to water molecules by the equatorial OH4 group. This difference is also apparent from Figure 7a, showing that OH4 forms significantly more intermolecular H-bonds with water in the case of β-d-fructopyranose. It is worth noting that for α-d-fructopyranose, the hydration preference also slightly shifts to the −y and −z directions due to significant intramolecular hydrogen bonding among the OH1–OH2, OH2–OH3, and OH1–OH3 pairs (cf. Figure 6c) on the +y side. Consequently, more water molecules are in the positive x and y directions in the hydrophobic sphere.

## Discussion

Our analysis revealed that the inherent conformational flexibility of the furanose ring—characterized by accessible transitions between multiple puckering states—enables a highly heterogeneous intramolecular H-bonding network. This diversity is driven primarily by the exocyclic OH group on the C6 arm and the two OH groups at the C1 position, which actively engage with other sites. In contrast, the more rigid pyranose tautomers preferentially form intramolecular bonds only between adjacent hydroxyl groups, while leaving the ring oxygen entirely uninvolved. Crucially, this exclusion extends to the intermolecular regime for both furanose and pyranose rings alike; in all cases, the ring oxygen was found to be the least effective acceptor for approaching water molecules—a behavior fundamentally rooted in the electronic structure, as these atoms consistently carry the smallest negative Bader charge. On the other hand, the OH sites engage in hydrogen bonding with water, revealing clear anomeric differences. Among the four studied systems, the β-pyranose emerges as the most efficient at H-bonding, with all of its OH sites participating nearly equally as both donors and acceptors. In contrast, the α-pyranose falls significantly behind. A similar, though more specific difference is observed for the furanoses, where the β-anomer outperforms the α-form because nearly all of its OH sites act as better acceptors. This enhanced H-bonding capacity of the β-anomers may reflect their higher macroscopic solubility.

Moreover, our analysis of the solvent environment extends beyond the first hydration shell, shedding light on the behavior of bulk-like water molecules that are not directly hydrogen-bonded to fructose. Across all four systems studied, we found that a four-fold coordination remains dominant for water molecules, regardless of whether they are directly bound to the fructose or interact exclusively with other water molecules. Importantly, no significant difference in these dipole moment values can be observed between molecules in the first H-bonding sphere and those further away. Notably, the dipole moment of these four-coordinated water molecules is significantly larger than that of the three-fold coordinated ones, which represent the second most frequent coordination state. Regarding the latter, we also revealed that the dipole moment of water molecules is affected not only by the number but also by the types of H-bonds formed, namely, the numbers of acceptor and donor bonds.

It should be emphasized that classical MD and AIMD provide complementary insights into carbohydrate solvation. While classical MD provides extensive conformational sampling over nanosecond time scales—making it highly reliable for statistical structural distributions—AIMD explicitly accounts for electronic polarization and dynamic charge transfer. Therefore, classical MD serves as the primary reference for long-range structural and overall statistical trends, whereas AIMD provides a physically accurate description of local, site-specific electronic interactions within the primary hydration shell.

In a broader context, beyond fundamental solvation properties, these localized hydration networks and asymmetric hydrogen-bonding properties play a pivotal role in macroscopic behavior. Specifically, the exceptionally strong sugar-water interactions contribute to the high aqueous solubility of sugars, while the structured hydration shell likely mediates molecular recognition within biological binding pockets, such as the human sweet taste receptor.^36^

## Conclusions

In summary, this work provides a comprehensive characterization of the complex hydrogen-bonding patterns of fructose. By extending our dual-scale MD/AIMD strategy to both pyranose and furanose tautomers of fructose, we have demonstrated that accurate electronic-level descriptions of polarization and charge transfer can be achieved without sacrificing statistical significance. Crucially, our findings reveal a tight coupling between intra- and intermolecular H-bonding networks, governed by the limited number of H-bond donor and acceptor sites provided by the hydroxyl groups and the ring oxygen. We demonstrate that when a specific site participates in an intramolecular bond, it is effectively rendered unavailable for cooperative interactions with the surrounding aqueous environment. By correlating the propensity of individual OH groups to form H-bonds with Bader charges of the carbon, oxygen, and hydroxyl sites, along with changes in water dipole moments, we provide a quantitative electronic-structure basis for understanding solvation properties. Furthermore, accounting for the local hydrophobic character of the hydration shell delivers an atomistic view of carbohydrate solvation that bridges structural flexibility and electronic-level polarization.

Finally, elucidating these site-specific, atomistic hydration patterns will provide a deeper understanding of how the ring puckering in furanoses, alongside the spatial orientation of the hydroxymethyl groups in both pyranose and furanose tautomers, influences carbohydrate behavior in biologically relevant processes, such as recognition and docking efficiency within metabolic enzymes, where the displacement of structured water molecules is a key driving force.

## Computational Details

### Classical Molecular Dynamics Simulation

Classical MD simulations were performed on fructose-water systems using the GROMACS 2023 software package.^37^ We carried out simulations of aqueous solutions of two five-membered ring (α- and β-d-fructofuranose) and two six-membered ring (α- and β-d-fructopyranose) isomers (cf. Figure 1). Our models consisted of one fructose molecule and 99 water molecules. The CHARMM36 all-atom interaction potential^38^ for fructose molecules and the SPC/E three-site water model^39^ were used. Periodic boundary conditions were applied, and the box sizes (L) were 1.51382 nm (α-d-fructofuranose), 1.49604 nm (β-d-fructofuranose), 1.51459 nm (α-d-fructopyranose), and 1.52055 nm (β-d-fructopyranose). This volume provides a spatial buffer exceeding 7.5 Å (L/2) from the solute heavy atoms. Since the first and second hydration shells extend to approximately 3.5 Å and 5.5 Å, respectively,^15^ this box size allows the second solvation shell to form naturally without boundary-induced artifacts. The Newtonian equations of motion were integrated via the leapfrog algorithm using a time step of 2 fs. The long-range forces were treated using the particle-mesh Ewald^40,41^ algorithm. The following simulation steps were applied after energy minimization: (1) 1 ns equilibration under NPT conditions using the Berendsen thermostat and barostat;^42^ (2) 1 ns equilibration under NVT conditions using the Berendsen thermostat at 298 K; (3) 600 ns production run under NVT conditions at 298 K using the Nosé-Hoover thermostat.^43,44^ Properties of these systems were obtained from independent configurations collected every 15,000 steps.

### Ab Initio Molecular Dynamics Simulation

Additionally, we performed AIMD simulations using the CP2K simulation package, version 3.9.^45–47^ The initial configuration for AIMD simulations was obtained from the classical MD simulations. By extracting initial configurations for AIMD from uncorrelated, thermally equilibrated snapshots of the multi-nanosecond classical MD trajectories, we ensure that the quantum-mechanical description of electronic properties, polarization, and directionality of hydrogen bonding is performed on statistically representative conformational states. This multi-scale strategy effectively mitigates the limited sampling window inherent to DFT-based AIMD simulations while preserving electronic-level accuracy.

The forces were calculated at the DFT/BLYP level of theory^48–50^ with the D3 dispersion corrections.^51^ The norm-conserving Goedecker-Teter-Hutter (GTH) pseudopotentials^52^ were used. Within the GPW (Gaussian and Plane Waves) method,^53^ the Kohn-Sham orbitals were expanded in a triple-zeta (TZ) basis set with double polarization functions (TZV2P), and a plane-wave basis with a cutoff of 300 Ry was used to represent the electron density. The NN50 smoothing procedure was applied to the electron density because Jonchiere et al.^54^ demonstrated that it produces well-converged simulation results. During each self-consistent field (SCF) cycle, the wave function coefficients were converged to a threshold of 10^–7^. In all simulations, the standard deviation of the total energy was about 10^–4^ Hartree. The production runs for each investigated system lasted at least 150 ps, following a 50 ps equilibration period in the NVT ensemble at 320 K. The time step was 0.5 fs, and the water molecule was handled as heavy water (D_2_O). This simulation setup has already been used in AIMD simulations of α-d-glucose, β-d-glucose, α-d-mannose, and α-d-galactose systems.^23^ The input parameters are detailed in the Supporting Information. For further analyses, three independent conformations were selected for fructofuranose systems and two for fructopyranose systems, ensuring that the hydroxymethyl group was in different states for each.

The Wannier center of the water molecule was calculated for every 200th time step (50 fs) in the trajectories from the production runs using an in-house Fortran code. Fractional net molecular charges were calculated by summing the fractional atomic charges, which were derived from a Bader-type analysis^55^ using the code of the Bader program downloaded from Henkelman’s group homepage.^56^ This method is an elegant application of Bader analysis, which determines non-overlapping volumes assignable to atoms based on the topological properties of the electron density. The program calculates the charges and dipole moments associated with the volumes of the atoms. The code calculated these properties using the CHGCAR (electron density) file produced by the CP2K code.^48^

Before presenting the results, it is essential to pay particular attention to the key structural and dynamical characteristics of the studied systems to verify whether they exhibit liquid behavior. All investigated properties, including the self-diffusion coefficient (Table S2), the partial radial distribution functions (Figure S7), and the reorientational correlation times (Table S3), were calculated for the water subsystems. The results confirmed that our systems are in a liquid state. Detailed analyses are available in the Supporting Information.

Puckering states were calculated using phase angle and puckering amplitude as outlined in ref 9. The complete pseudorotational wheel illustrating the different puckering states of d-fructofuranoses is shown in Figure S5. These calculations, together with dihedral angles, H-bond numbers, and hydration sphere calculations, were performed using in-house code.

## Supplementary Material



## Notes

Details of 2D probability distributions of dihedral angles; pseudorotational wheel for d-fructofuranoses; 2D density distribution functions; radial distribution functions and angular distribution functions; H-bond properties; electronic properties of fructose; electronic properties of water; hydration properties; input file for CP2K simulations; self-diffusion coefficients and reorientational dynamics (PDF)
