# Supplementary material for: Unraveling the Complex Hydrogen-Bonding and Solvation Patterns of Fructose Tautomers via Complementary AIMD and Classical MD Simulations
Source: ACS Phys Chem Au. 2026 Sep 8;6(5):1372–86. doi: 10.1021/acsphyschemau.6c00122 (PMC13614054; doi:10.1021/acsphyschemau.6c00122)
Supplement: Supplementary file 1 [file pg6c00122_si_001.pdf]

Supporting Information

for

Unraveling the Complex Hydrogen-Bonding and Solvation Patterns of Fructose Tautomers via Complementary AIMD and Classical MD Simulations

Imre Bakó[1]*, Szilvia Pothoczki[2]*

[1]HUN-REN Research Centre for Natural Sciences, H-1117 Budapest, Magyar tudósok körútja 2., Hungary

[2]HUN-REN Wigner Research Centre for Physics, H-1121 Budapest, Konkoly-Thege M. út 29-33., Hungary

*Correspondence: bako.imre@ttk.hu, pothoczki.szilvia@wigner.hu

Content:

1. 2D probability distributions of dihedral angles
2. Pseudorotational wheel for D-fructofuranoses
3. 2D density distribution functions
4. Radial distribution functions and angular distribution functions
5. H-bond properties
6. Electronic properties of fructose
7. Electronic properties of water
8. Hydration properties
9. Input file for CP2K simulations
10. Self-diffusion coefficients
11. Re-orientational dynamics

1. 2D probability distributions of dihedral angles

1.1 α-D-fructofuranose

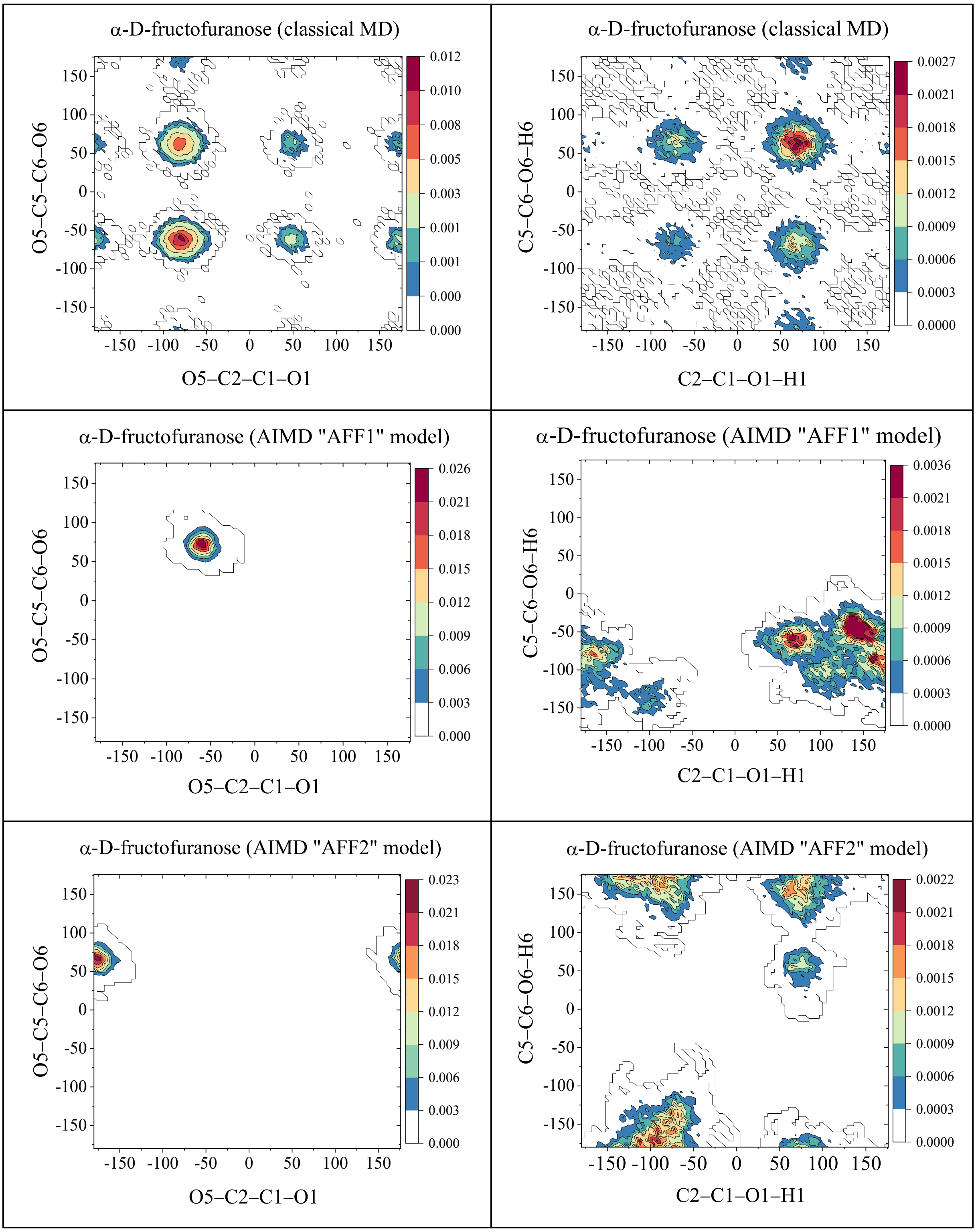

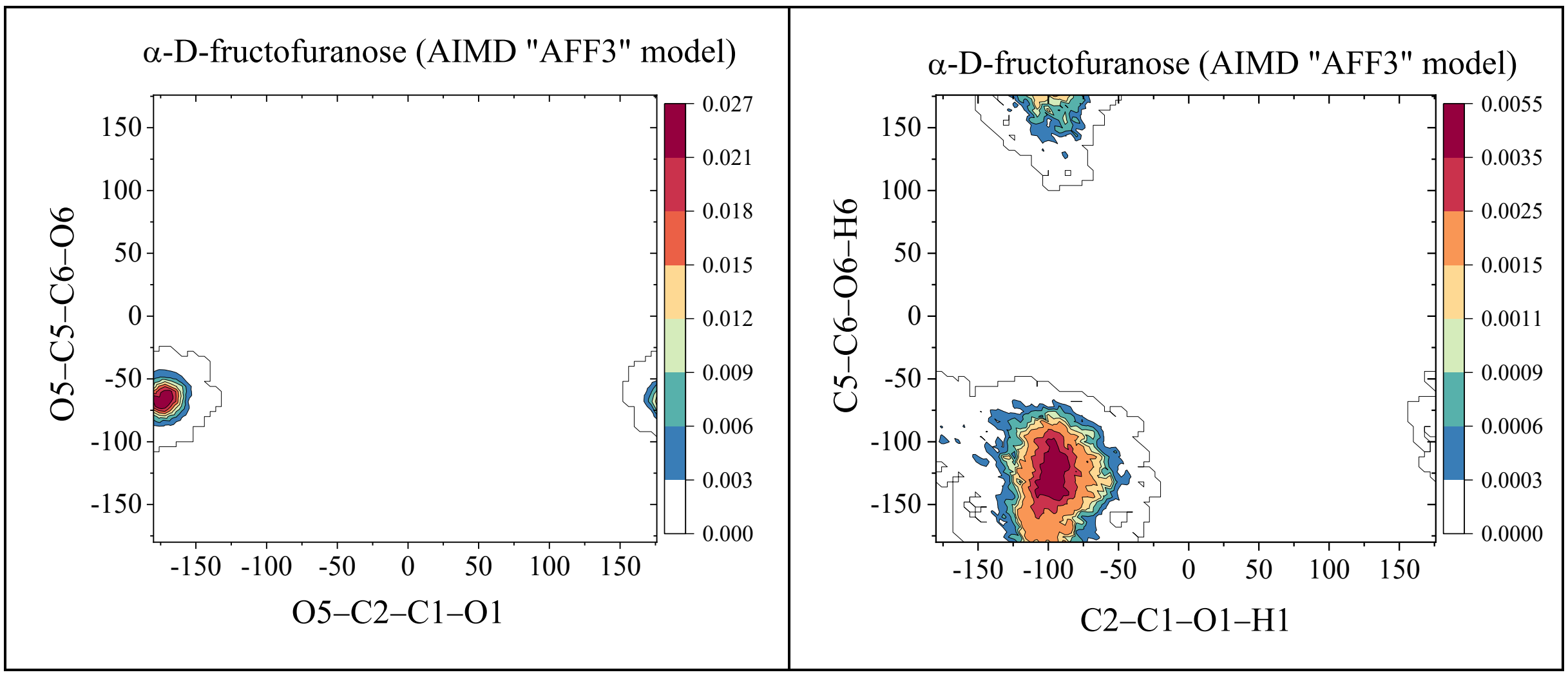

Figure S1. 2D probability distributions of dihedral angles (deg) obtained from classical and ab initio MD simulations for α-D-fructofuranose.

1.2 β-D-fructofuranose

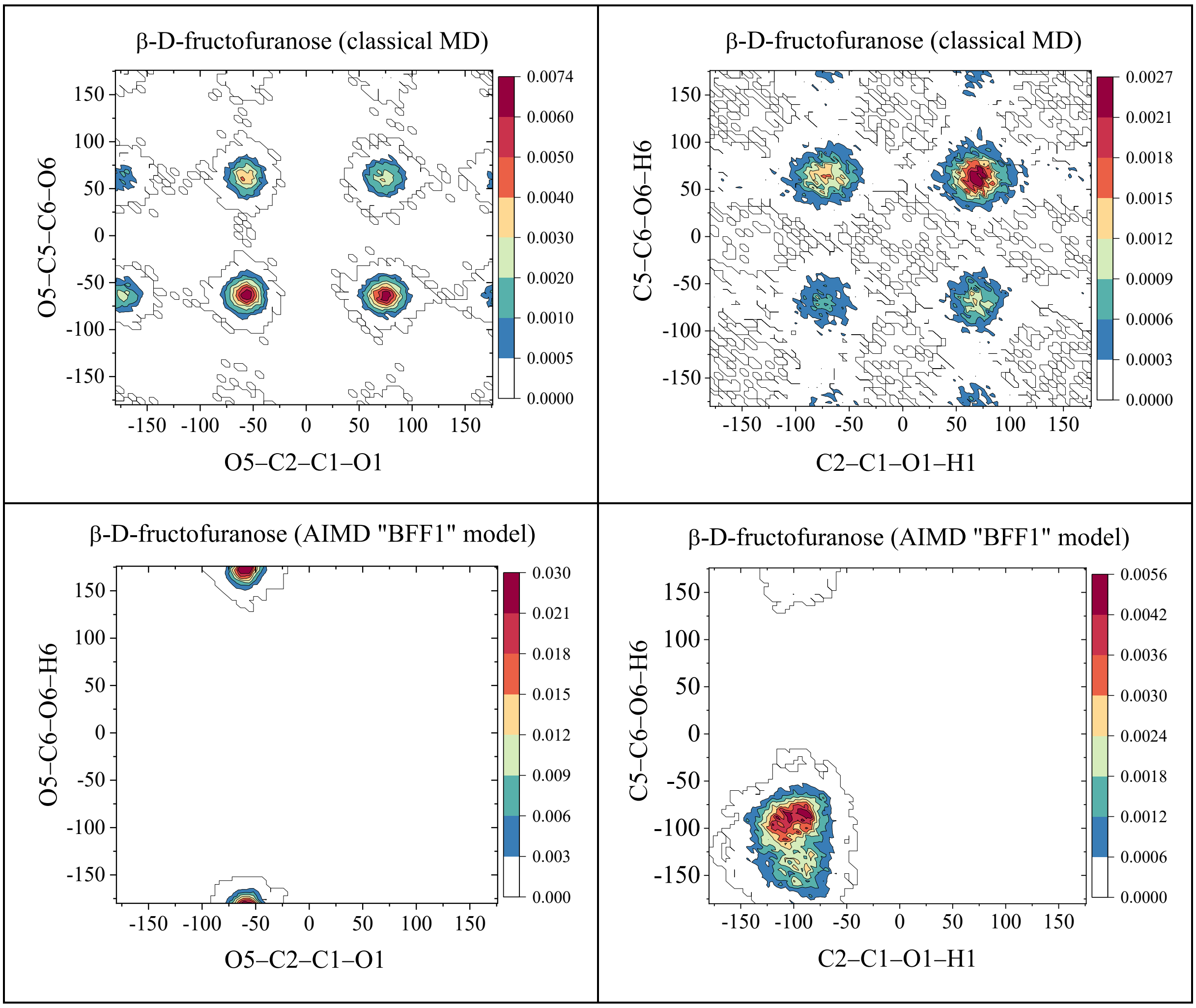

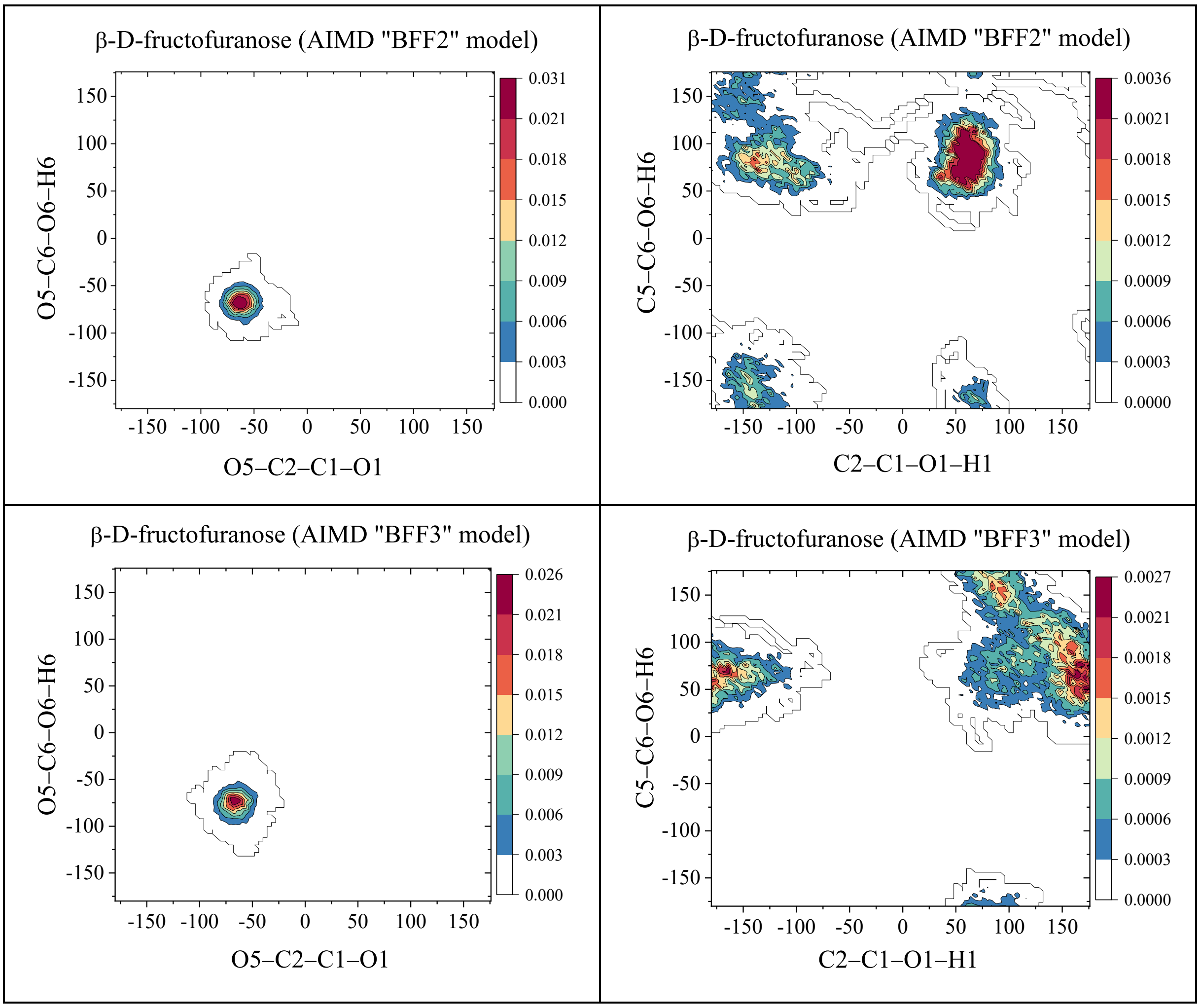

Figure S2. 2D probability distributions of dihedral angles (deg) obtained from classical and ab initio MD simulations for β-D-fructofuranose.

1.3 α-D-fructopyranose

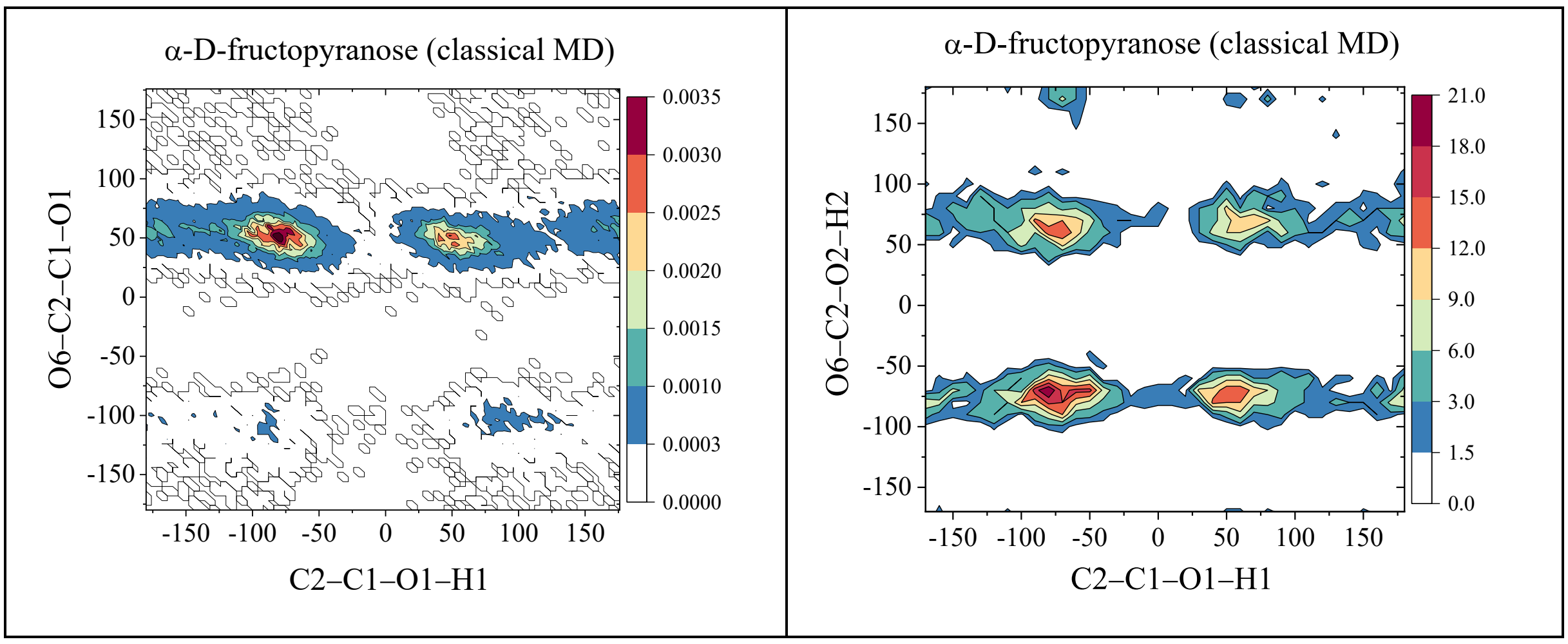

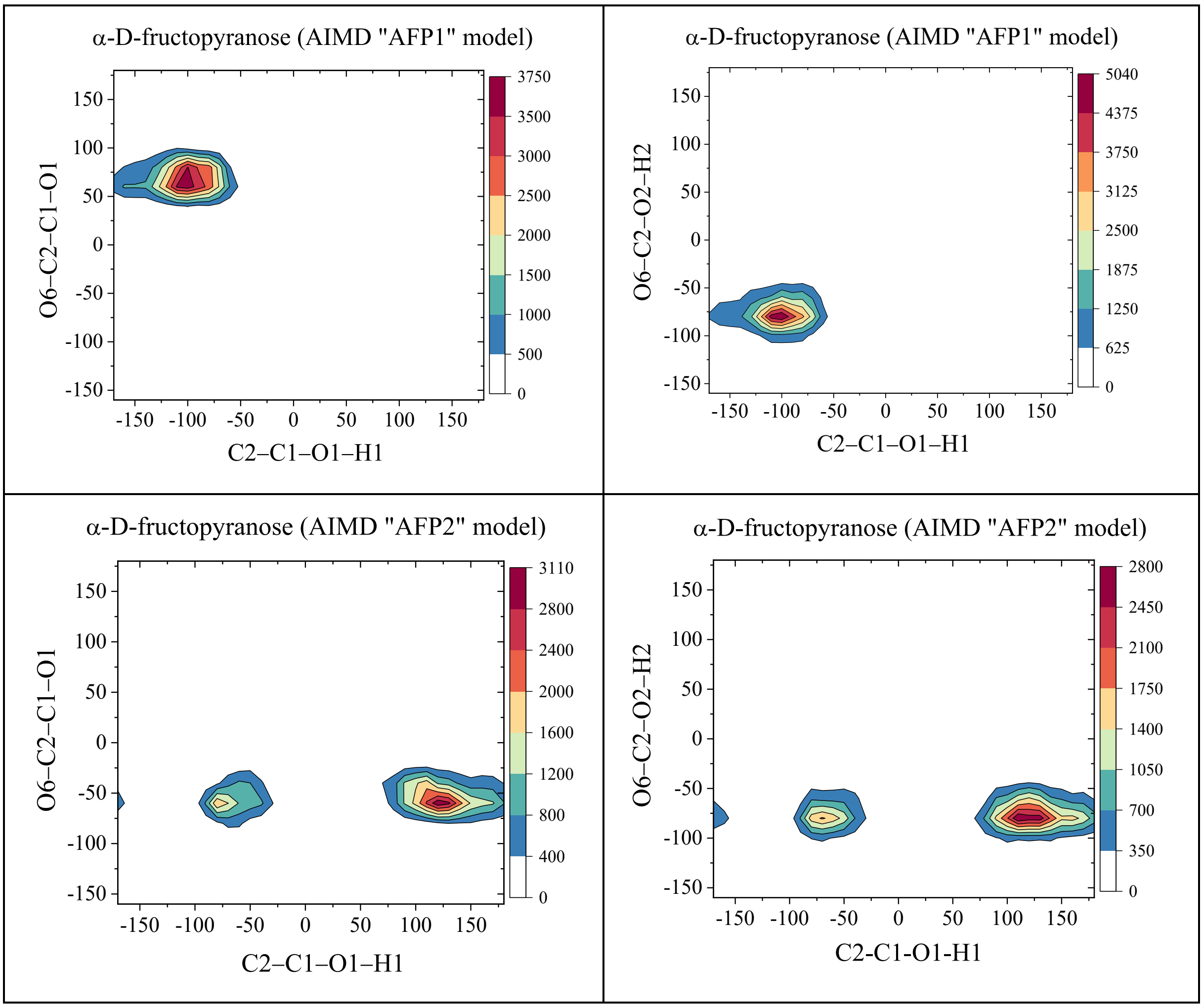

Figure S3. 2D probability distributions of dihedral angles (deg) obtained from classical and ab initio MD simulations for α-D-fructopyranose.

1.4 β-D-fructopyranose

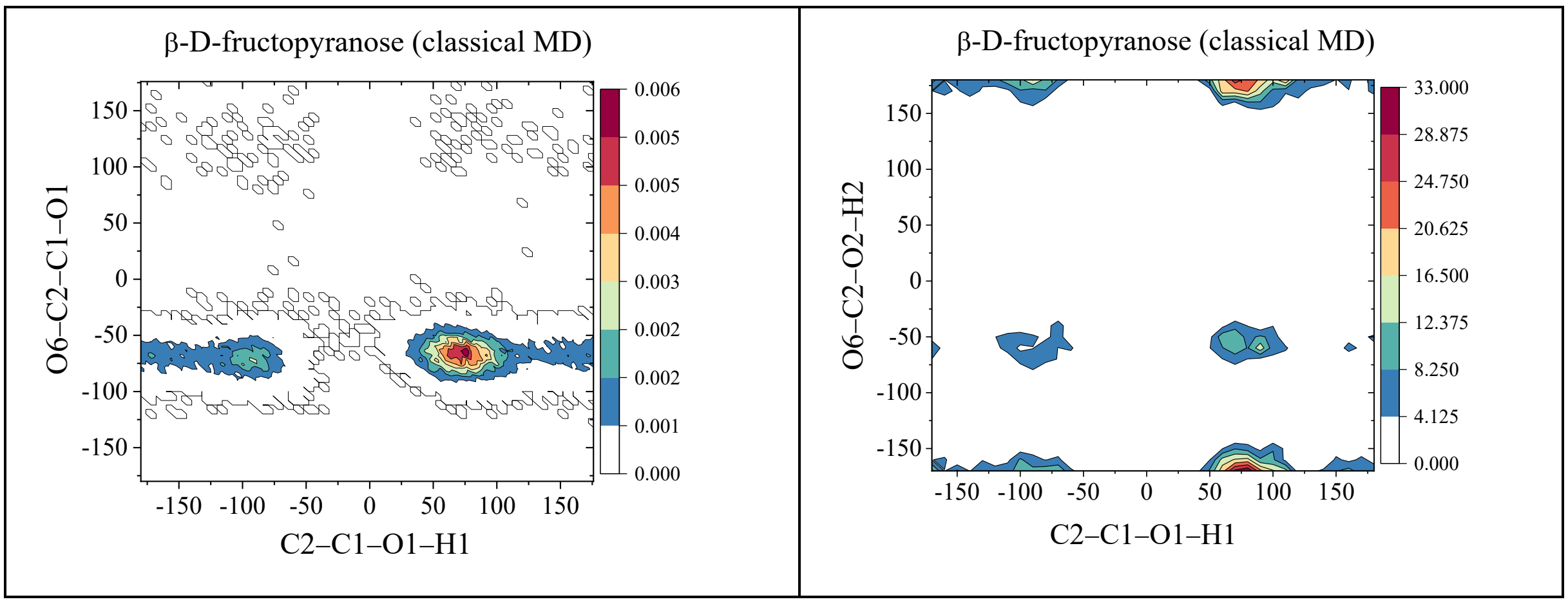

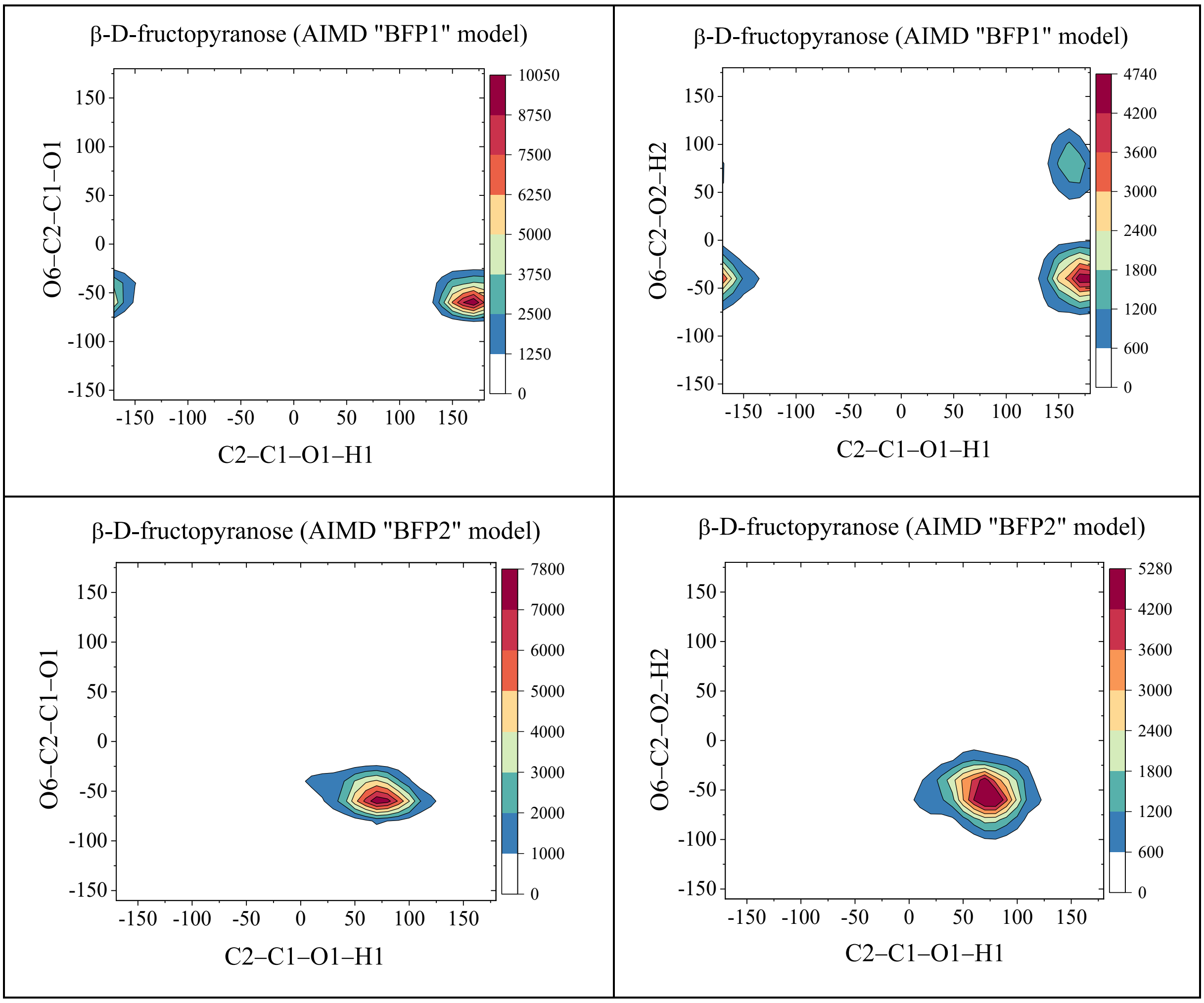

Figure S4. 2D probability distributions of dihedral angles (deg) obtained from classical and ab initio MD simulations for β-D-fructopyranose.

2. Pseudorotational wheel for D-fructofuranoses

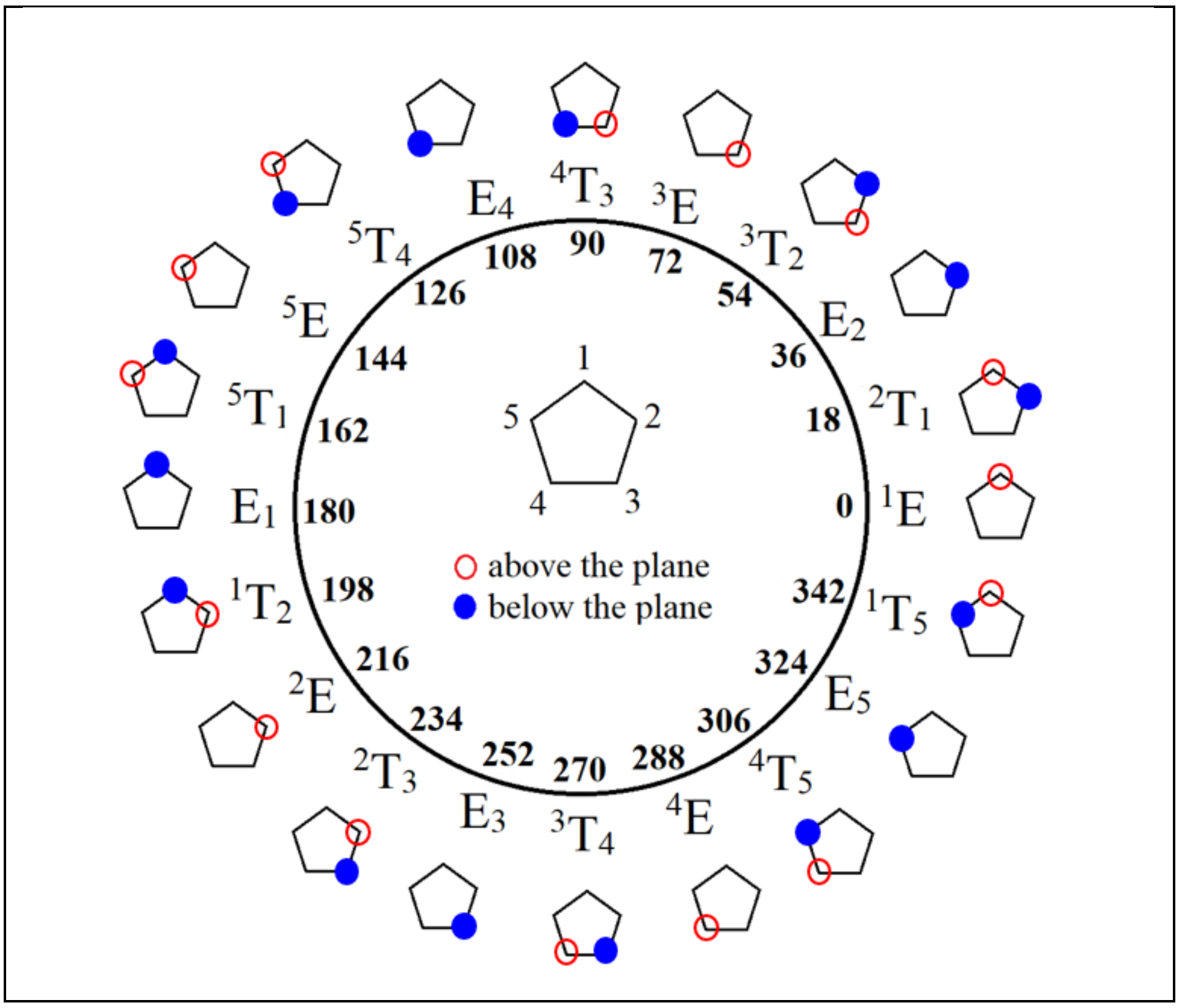

Figure S5. Pseudorotational wheel depicting the different puckering states for D-fructofuranose systems.

3. 2D density distribution functions

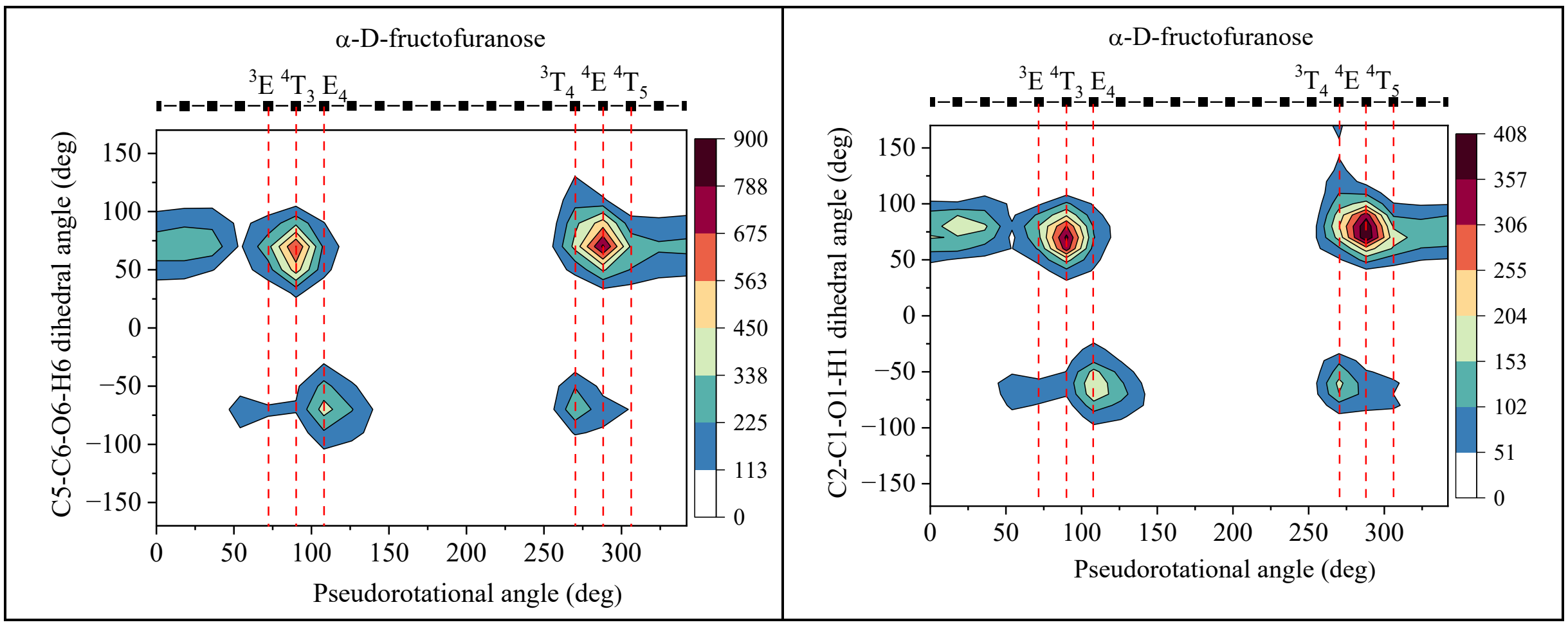

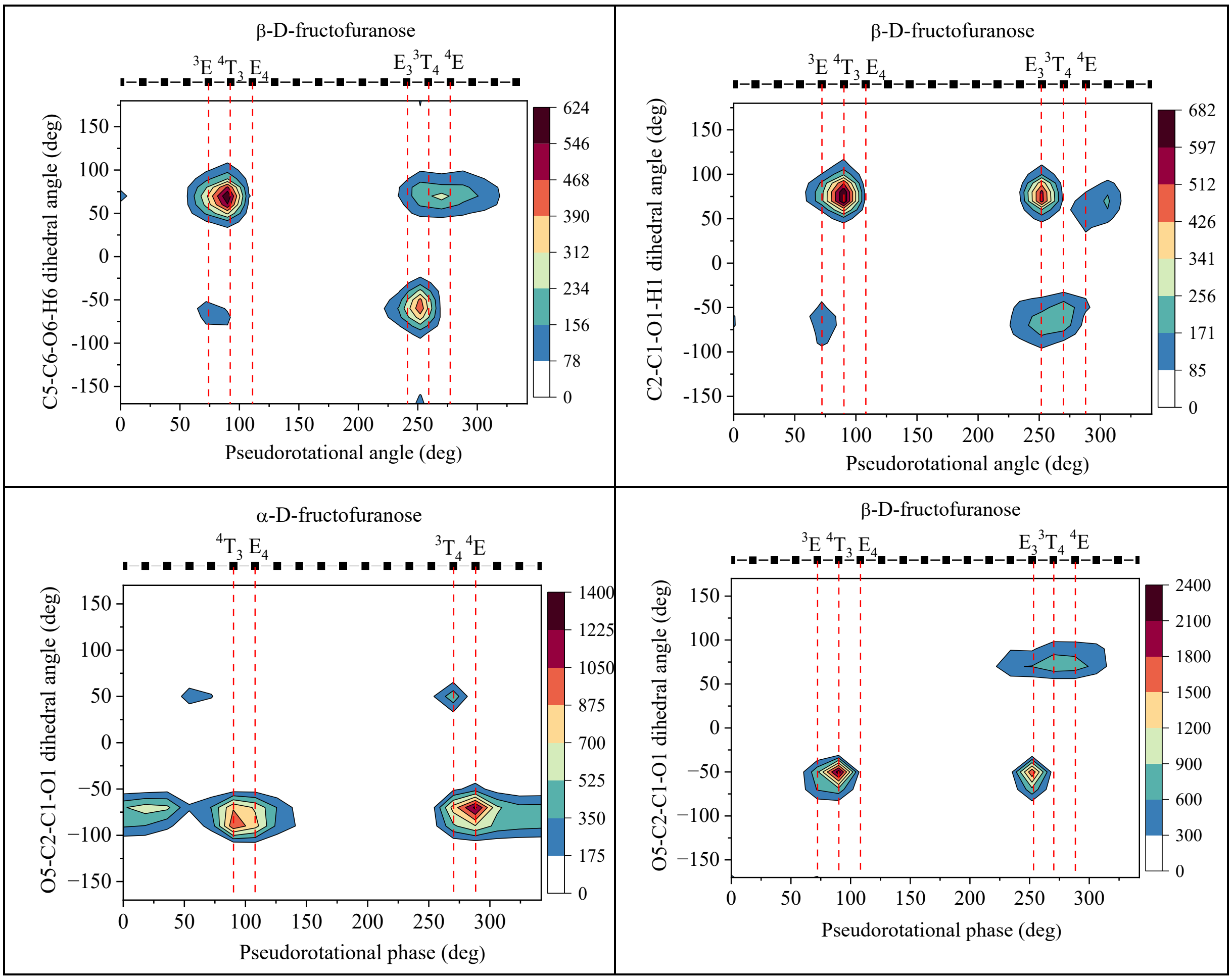

Figure S6. 2D density distribution functions of the pseudorotation phase angle and dihedral angles connecting to the hydroxymethyl group calculated from classical MD.

4. Radial distribution functions and angular distribution functions

The O-O and O-H radial distribution functions (rdf) were calculated for water subsystems from both classical and ab initio MD models. Figure S7 presents the results for all studied systems (both classical MD and ab initio models for fructofuranose and fructopyranose systems). Both cases (O-O and O-H) are very similar (in terms of the positions of the maxima and minima) to the corresponding functions of the SPC/E water model. However, in AIMD models, the peak intensity of O-O rdf is lower at minimum values and higher at maximum values, respectively, compared to the SPC/E systems. This suggests that the systems are slightly below 320 K but still in a liquid state. The first minimum (2.5 Å) of the O-H rdf was used for H-bond definition.

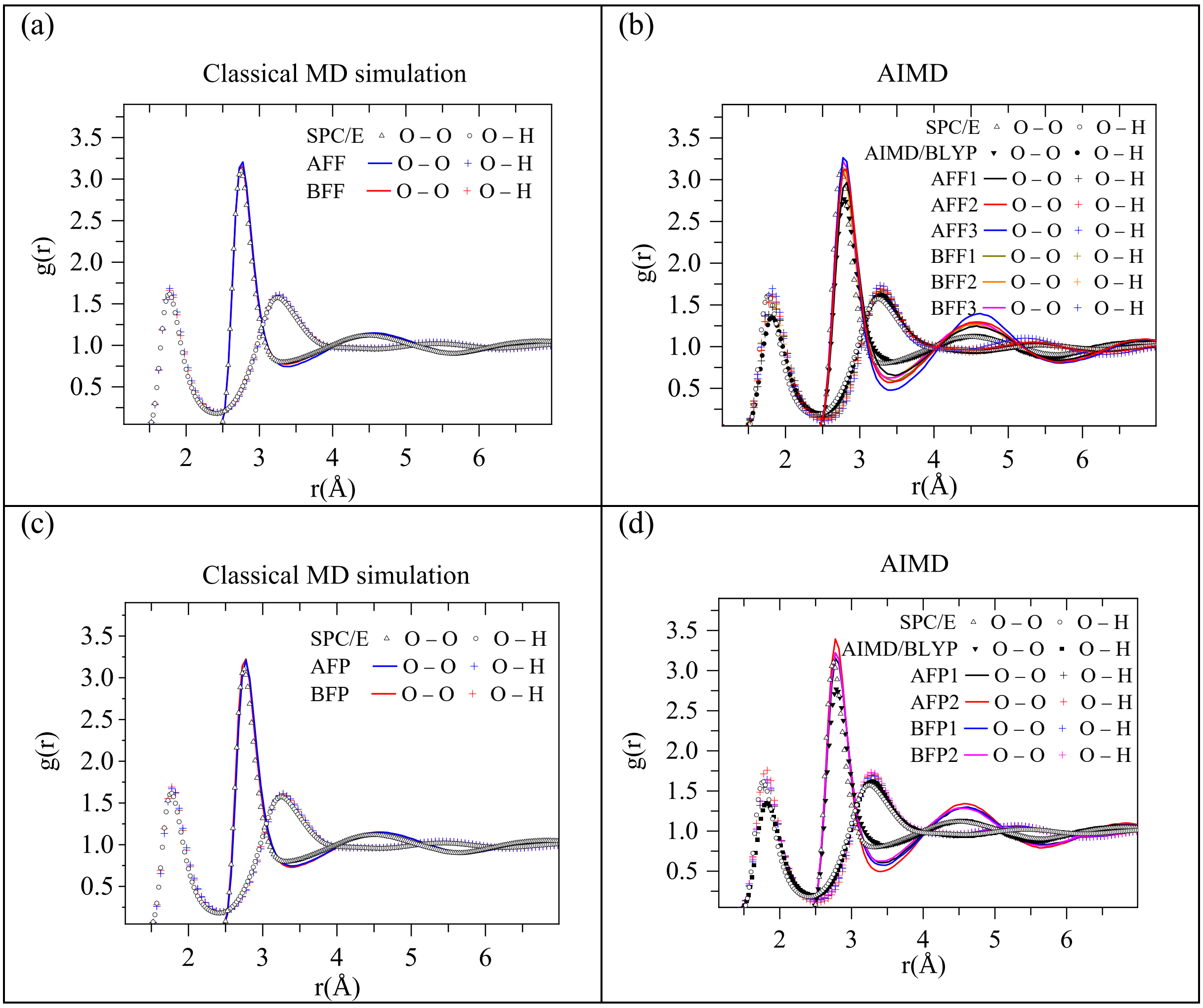

Figure S7. The O-O and O-H radial distribution functions of water subsystems calculated from a) classical MD models of fructofuranose systems, b) ab initio MD models of fructofuranose systems, c) classical MD models of fructopyranose systems, d) ab initio MD models of fructopyranose systems. AFF: α-D-fructofuranose, BFF: β-D-fructofuranose, AFP: α-D-fructopyranose, BFP: β-D-fructopyranose.

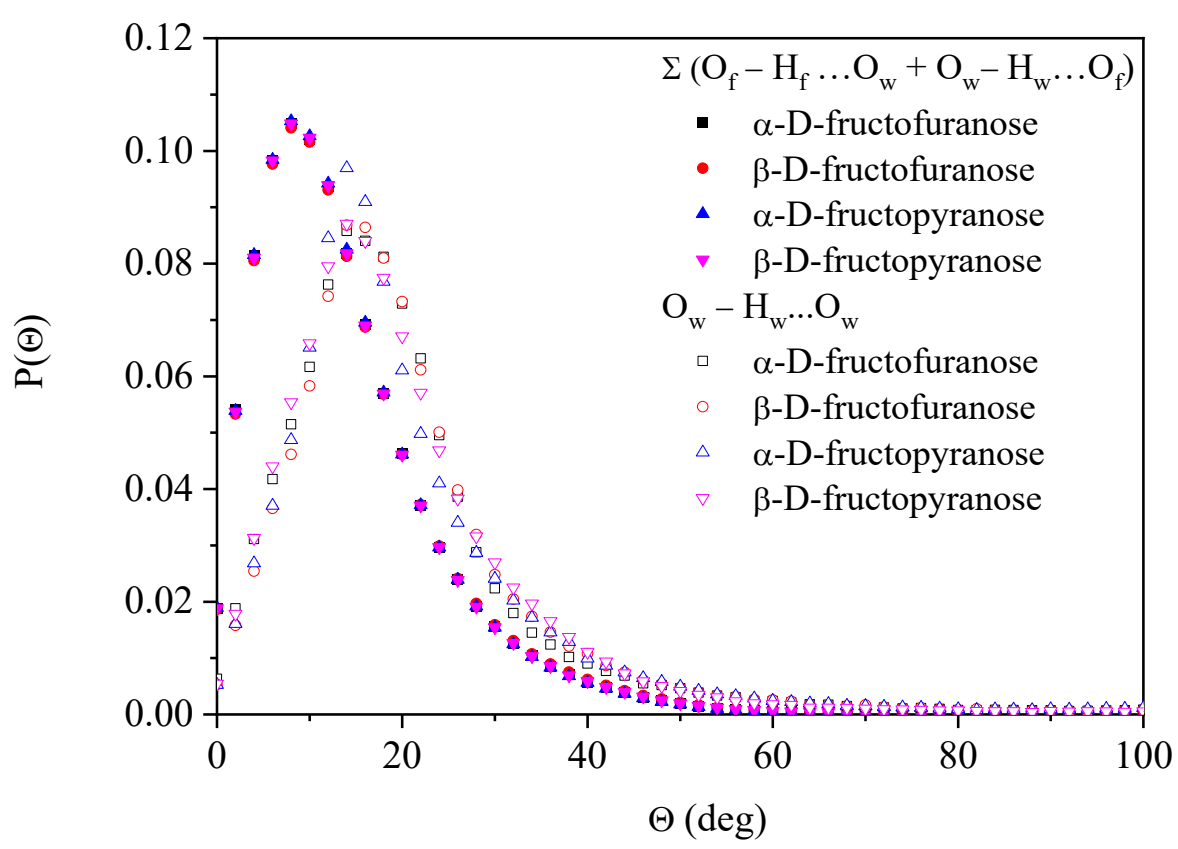

Figure S8. Angular distributions of intermolecular O-H…O angles (Θ) based on classical MD simulations.

5. *H-bond properties*

Table S1. Intramolecular H-bond probabilities calculated with the more strict definition: O-H…O < 30° and r(O···H) < 2.5 Å from classical MD models. AFF: α-D-fructofuranose, BFF: β-D-fructofuranose, AFP: α-D-fructopyranose, BFP: β-D-fructopyranose.

		AFF	BFF	AFP	BFP
1	2	0.010	0.003	0.004	0.000
1	3	0.039	0.001	0.360	0.000
1	4	0.001	0.021	0.000	0.000
1	5	0.000	0.000	0.000	0.000
1	6	0.069	0.018	0.000	0.000
2	3	0.001	0.003	0.608	0.000
2	4	0.026	0.001	0.000	0.000
2	5	0.000	0.000	0.000	0.000
2	6	0.001	0.047	0.000	0.000
3	4	0.000	0.000	0.000	0.000
3	5	0.000	0.000	0.000	0.000
3	6	0.130	0.118	0.000	0.000
4	5	0.000	0.000	0.000	0.000
4	6	0.000	0.000	0.000	0.000
5	6	0.000	0.000	0.000	0.000

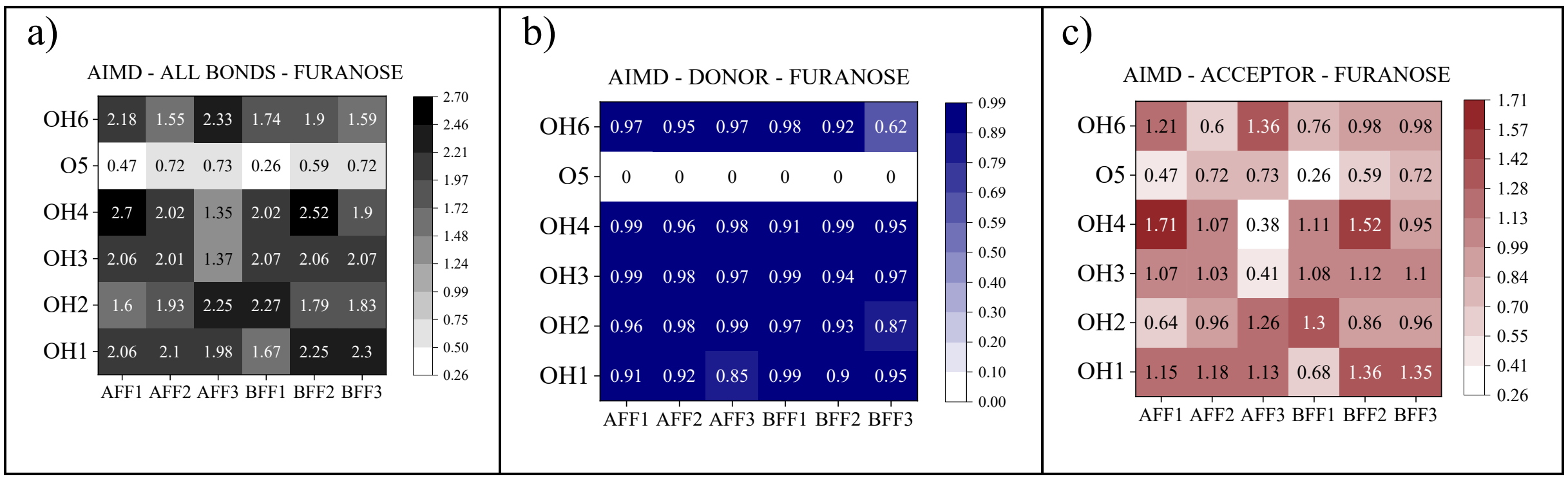

Figure S9. The average number of H-bonds together with the acceptor and donor H-bond numbers. a) all H-bonds for fructofuranose systems from AIMD, e) donor type H-bonds for fructofuranose systems from AIMD, and c) acceptor type H-bonds for fructofuranose systems from AIMD.

6. *Electronic properties of fructose molecules*

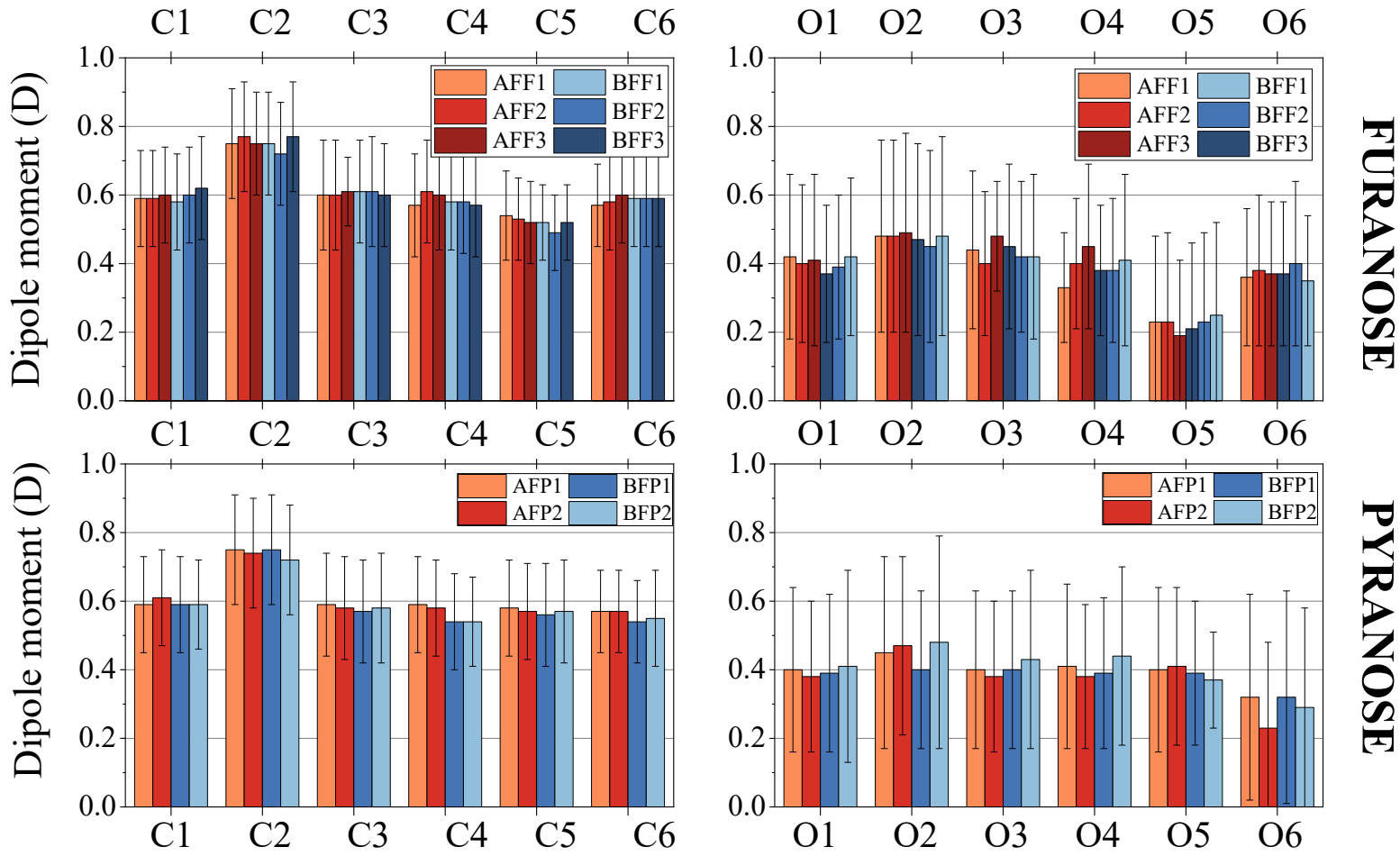

Figure S10. Dipole moment (D) of the C and O sites of the fructose molecule in the solutions based on AIMD simulations. Note that the dipole moments were derived from a Bader-type analysis[1] using the code of the Bader program downloaded from Henkelman's group homepage.[2]

7. *Electronic properties of water*

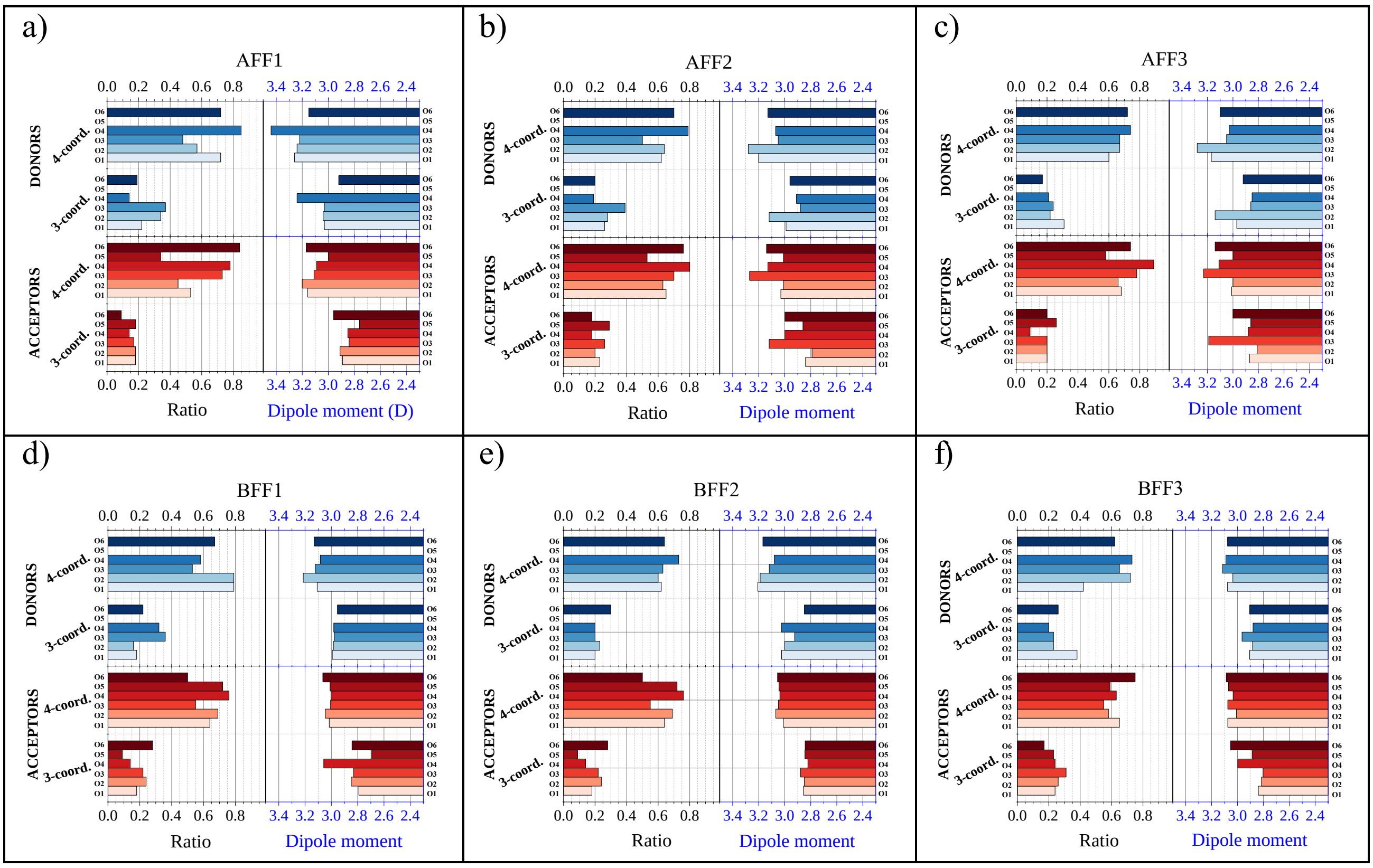

---

[1] Henkelman, G.; Arnaldsson, A.; Jónsson, H. A fast and robust algorithm for Bader decomposition of charge density. Comput. Mater. Sci. 2006, 36, 354-360. https://doi.org/10.1016/j.commatsci.2005.04.010

[2] Henkelman Group. Bader Charge Analysis Code. https://theory.cm.utexas.edu/henkelman/code/bader/ (accessed 2026-08-26).

Figure S11. Properties of three- and four-coordinated water molecules around the fructose molecule based on AIMD simulation. (Note that the acceptor or donor nature refers to the fructose molecules.) (a) Model 1 of α-D-fructofuranose, (b) Model 2 of α-D-fructofuranose, (c) Model 3 of α-D-fructofuranose, (d) Model 1 of β-D-fructofuranose, (e) Model 2 of β-D-fructofuranose, and (f) Model 3 of β-D-fructofuranose.

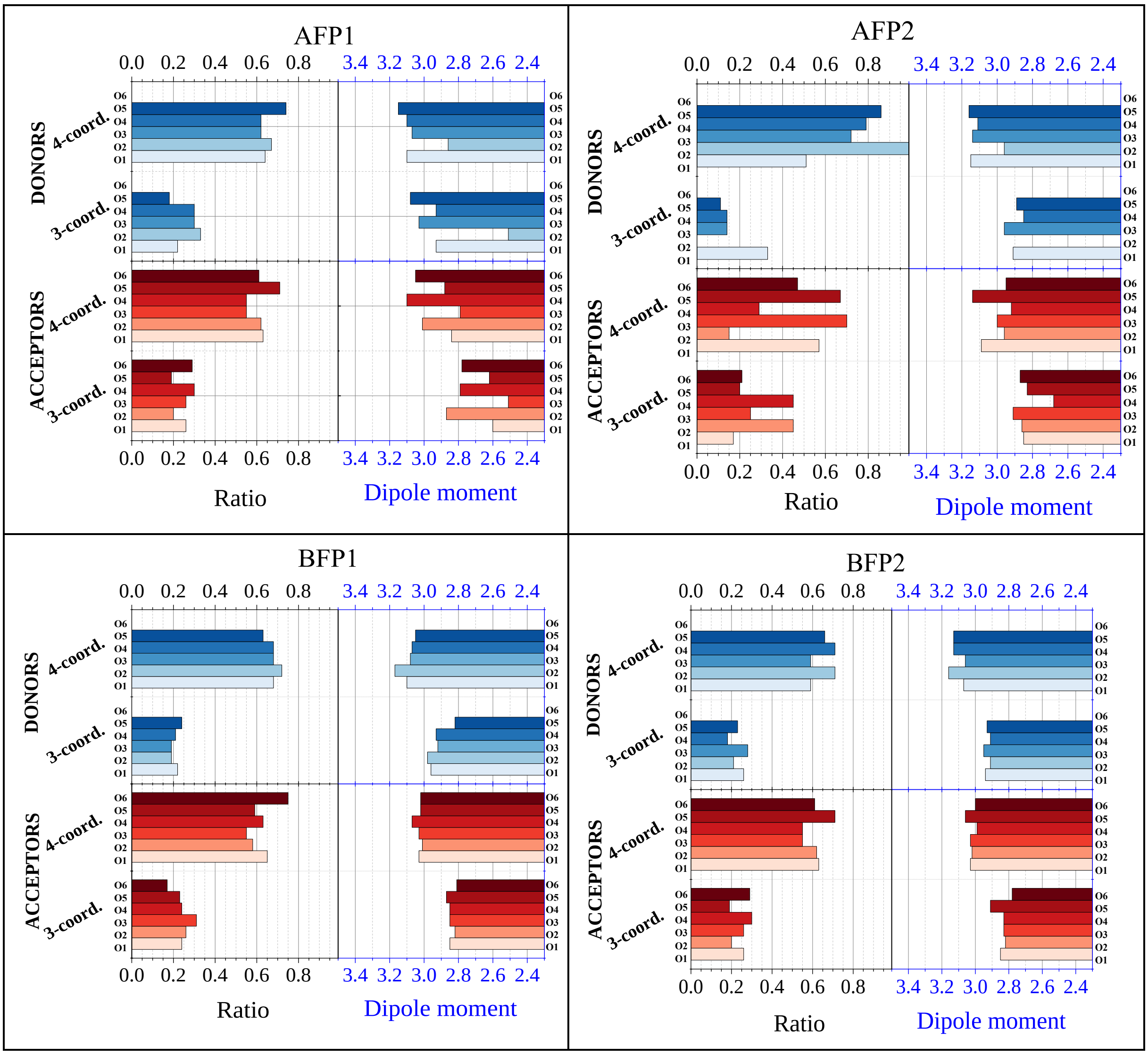

Figure S12. Properties of three- and four-coordinated water molecules around the fructose molecule based on AIMD simulation. (Note that the acceptor or donor nature refers to the fructose molecules.) (a) Model 1 of α-D-fructopyranose, (b) Model 2 of α-D-fructopyranose, (c) Model 3 of α-D-fructopyranose, (d) Model 1 of β-D-fructopyranose, (e) Model 2 of β-D-fructopyranose, and (f) Model 3 of β-D-fructopyranose.

8. Hydration properties

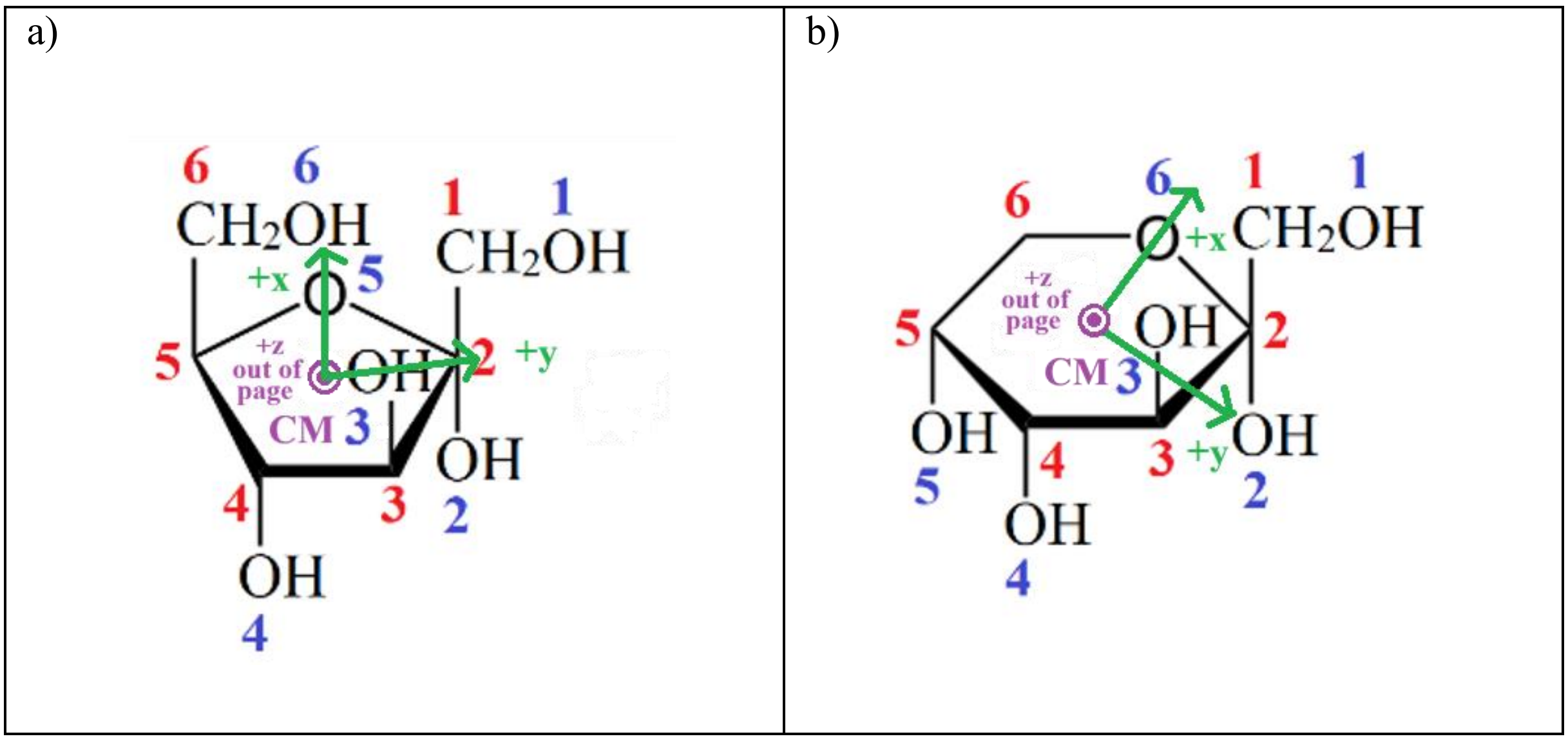

Figure S13. A schematic representation of the coordinate system for calculations of hydration sphere properties. a) for α-D-glucofuranose and b) for α-D-glucopyranose. CM: Center of mass. (The coordinate system for the beta anomers (D-fructofuranose and D-fructopyranose) is the same as for the alpha form.

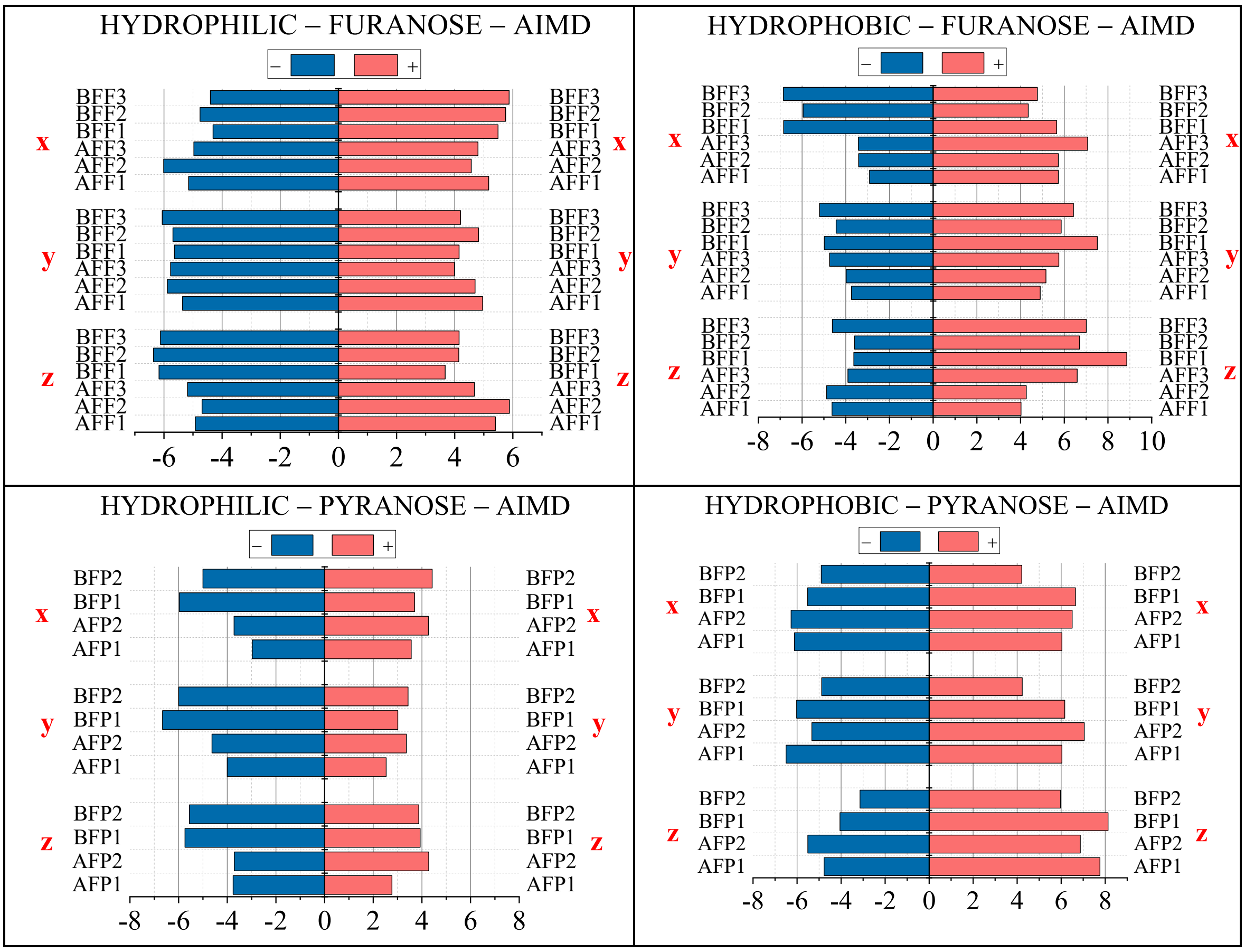

Figure S14. The number of hydrophobic and hydrophilic H-bonded water molecules above and below the XY, YZ, and XZ molecular plane of fructose molecules based on AIMD simulation.

9. Input file for CP2K simulations

```
&MOTION

  &MD
    ENSEMBLE  NVT
    STEPS ${steps}
    TIMESTEP   ${timestep}
    TEMPERATURE    ${temperature}

      &THERMOSTAT
     &NOSE
            LENGTH           3
            TIMECON    1.6680000000000000E+01
     &END NOSE
    &END THERMOSTAT
    &AVERAGES  T
    &END AVERAGES
  &END MD
  &PRINT
 &TRAJECTORY  SILENT
   FILENAME ${projectname}
   FORMAT  XMOL
  &EACH
         MD          2
  &END EACH
 &END TRAJECTORY
&VELOCITIES  SILENT
   FILENAME ${projectname}
   FORMAT  XMOL
  &EACH
```

```
                MD              2
     &END EACH
   &END VELOCITIES

      &END PRINT
&END MOTION
&FORCE_EVAL
  METHOD  QS
  &DFT
  BASIS_SET_FILE_NAME   ${LIB}/BASIS_MOLOPT
   POTENTIAL_FILE_NAME   ${LIB}/GTH_POTENTIALS
              CHARGE    ${charge}
              &QS
              &END QS
&LOCALIZE
 &PRINT
 &WANNIER_CENTERS
 IONS+CENTERS
  &EACH
                           MD  200
                      &END EACH
                  &END WANNIER_CENTERS
&END PRINT
 &END LOCALIZE

              &MGRID
               NGRIDS             5
               CUTOFF     3.0000000000000000E+02
              &END MGRID
```

```
&SCF
        SCF_GUESS ATOMIC
        EPS_SCF 1.E-7
        MAX_SCF_HIST 100

        &OT ON
             MINIMIZER  DIIS
             PRECONDITIONER  FULL_ALL
             STEPSIZE  0.08
        &END OT

        &OUTER_SCF
            OPTIMIZER  DIIS
            EPS_SCF 1.0E-7
&END OUTER_SCF

&END SCF

&XC
        &XC_FUNCTIONAL BLYP
        &END XC_FUNCTIONAL
        &XC_GRID
         XC_SMOOTH_RHO  NN50
        &END XC_GRID
&KIND H
          MASS 2.0
          BASIS_SET  TZV2P-MOLOPT-GTH-q1
          POTENTIAL  GTH-BLYP-q1
        &END KIND
        &KIND O
```

```
  BASIS_SET TZV2P-MOLOPT-GTH-q6
  POTENTIAL GTH-BLYP-q6
&END KIND
&KIND C
  BASIS_SET TZV2P-MOLOPT-GTH-q4
  POTENTIAL GTH-BLYP-q4
&END KIND
```

10. Self-diffusion coefficients

The self-diffusion coefficient (D) was estimated using the Einstein-Smoluchowski relation, from the mean squared displacements of the centers of mass of water molecules – essentially reflecting the mean square displacement of the oxygen atoms:

$$D = \lim_{t\to\infty} \frac{1}{6Nt} \left\langle \sum_{i=1}^{N} \left(r_i(t) - r_i(0)\right)^2 \right\rangle \quad (1)$$

where $r_i(t)$ and $r_i(0)$ are the positions of the centers of mass of water molecules at time t and 0, respectively, and the <…> denotes an ensemble average.

The self-diffusion coefficients of water molecules based on ab initio and classical MD simulations are presented in Table S2 for each studied system. It is important to note that the calculations from the AIMD simulations were applied to heavy water (D_2O), accounting for finite-size effects and using a shear viscosity of 1.107 cP (centipoise) for pure liquid D_2O.[3] These calculated values fall into the order of magnitude of the experimental diffusion coefficient of pure D_2O liquid ($0.1872 \cdot 10^{-8}$ m^2/s).[4] In the case of classical MD models, the self-diffusion coefficient of water molecules was calculated from the mean-square displacement (MSD) using the gmx msd tool provided within the GROMACS software package, according to the Einstein relation. These calculated values are in good agreement with the self-diffusion coefficient of the SPC/E water model ($0.25 \cdot 10^{-8}$ m^2/s). [5,6]

Table S2. Calculated self-diffusion coefficients for water molecules from ab initio and classical MD simulations for the studied models.

Systems	Self-diffusion coefficients (10^{-8} m^2/s)			
	classical MD	AIMD Model 1	AIMD Model 2	AIMD Model 3
α-D-fructofuranose	0.214±0.002	0.12±0.002	0.14±0.002	0.12±0.002
β-D-fructofuranose	0.218±0.024	0.18±0.002	0.16±0.002	0.16±0.002
α-D-fructopyranose	0.205±0.031	0.11±0.002	0.1±0.002	
β-D-fructopyranose	0.215±0.030	0.12±0.002	0.11±0.002	

[3] Harris, K. R.; Woolf, L. A. Temperature and Volume Dependence of the Viscosity of Water and Heavy Water at Low Temperatures. J. Chem. Eng. Data 2004, 49 (4), 1064–1069. https://doi.org/10.1021/je049918m.
[4] Mills, R. Self-Diffusion in Normal and Heavy Water in the Range 1–45 °C. J. Phys. Chem. 1973, 77 (5), 685–688. https://doi.org/10.1021/j100624a025.
[5] Vega, C.; Abascal, J. L. F. Simulating Water with Rigid Non-Polarizable Models: A Review. Phys. Chem. Chem. Phys. 2011, 13 (44), 19663–19688. https://doi.org/10.1039/c1cp21114c.
[6] Berendsen, H. J. C.; Grigera, J. R.; Straatsma, T. P. The Missing Term in Effective Pair Potentials. J. Phys. Chem. 1987, 91 (24), 6269–6271. https://doi.org/10.1021/j100308a038.

11. Re-orientational dynamics

Re-orientational dynamics can be described using the autocorrelation functions:

$$C_l(t) = \langle P_l(\underline{e}(t) \cdot \underline{e}(0) \rangle \quad (2)$$

where $\underline{e}(t)$ is the unit vector along a well-defined molecular axis (O_w-H_w vector, perpendicular to the HOH water molecular plane) and P_l is the l-th Legendre polynomial. Here, the first and second-order Legendre polynomials were used. The decay time of these autocorrelation functions, <τ>, is estimated by computing the integral of $C_l(t)$ with respect to time, that is:

$$\tau = \int_0^{\infty} C_l(t)dt \quad (3)$$

The calculated reorientational times are found in Table S3.

The integrals were carried out numerically up to 15 ps. The contribution from the long tail part of $C_l(t)$ functions was calculated using a fitting procedure, and the integral was carried out based on the fitting value of exponential decay.

The experimentally measured reorientational time, corresponding to the P_2(OH) autocorrelation function, obtained from NMR experiments, is 1.7-2.6 ps.[7] It is worth noting that this value significantly increases as the temperature decreases.[7] The results proved that the calculated dynamical properties are not significantly larger than the corresponding experimental values.

Table S3. The characteristic time of reorientation of OH vectors of water molecules in the studied monosaccharide solutions (in ps) from AIMD.

Systems	Model 1	Model 2	Model 3
	P_1 (OH) (ps)		
α-D-fructofuranose	14.0	9.6	11.8
β-D-fructofuranose	11.3	15.4	10.9
α-D-fructopyranose	11.4	14.6	
β-D-fructopyranose	11.6	12.4	
	P_2 (OH) (ps)		
α-D-fructofuranose	9.9	5.2	7.1
β-D-fructofuranose	6.7	7.5	6.5
α-D-fructopyranose	6.8	10.5	
β-D-fructopyranose	7.3	7.5	
	P (HOH plane) (ps)		
α-D-fructofuranose	12.2	7.4	9.9
β-D-fructofuranose	9.3	11.0	8.8
α-D-fructopyranose	8.9	13.0	
β-D-fructopyranose	9.3	9.8	

[7] Piskulich, Z. A.; Thompson, W. H. The Activation Energy for Water Reorientation Differs Between IR Pump-Probe and NMR Measurements. *J. Chem. Phys.* **2018**, *149* (16), 164504. https://doi.org/10.1063/1.5050203.
